# Comparative study of immunohematological tests with canine blood samples submitted for a direct antiglobulin (Coombs’) test

**DOI:** 10.1186/s40575-021-00107-0

**Published:** 2021-10-13

**Authors:** Nadine Idalan, Johanna O. Zeitz, Corinna N. Weber, Elisabeth Müller, Urs Giger

**Affiliations:** 1 Vetsuisse Faculty, University of Zürich, Winterthurerstrasse 260, 8057 Zürich, Switzerland; 2 Laboklin GmbH&Co.KG, Steubenstrasse 4, 97688 Bad Kissingen, Germany; 3 Section of Medical Genetics, University of Pennsylvania, 3900 Delancey St, Philadelphia, PA 19104 USA

**Keywords:** Autoagglutination, Spherocytes, Erythrocytes, Dog, Canine, Immune-mediated hemolytic anemia

## Abstract

**Background:**

A 2019 ACVIM consensus statement on diagnostics for immune-mediated hemolytic anemia (IMHA) in dogs made testing recommendations. As data on the performance of immunohematological tests was lacking, we undertook a comparative analysis.

**Material and methods:**

Anticoagulated blood samples from 126 dogs suspected of having IMHA submitted to a diagnostic veterinary laboratory for a routine direct antiglobulin test (DAT) and from 28 healthy control dogs were evaluated for spherocytosis and autoagglutination before and after three saline washes. Samples were also subjected to different DATs: a gel minitube and an immunochromatographic strip kit used in clinics; neutral gel column cards, microtiter plates (at 4°, 22°, and 37°C), capillary tubes, and flow cytometry used in laboratories.

**Results:**

Samples from healthy dogs yielded negative results with all immunodiagnostic tests. Among the 126 samples submitted for DAT 67 were positive by a DAT utilizing microtiter plates with goat anti-dog antiglobulin DAT at 22°C. Notably, DAT results were comparable and consistent across all evaluated methods regardless of antiglobulin and temperature used. DAT+ dogs were more severely anemic and more likely to have erythroid regeneration compared to DAT- dogs. Macroscopic agglutination in tubes or on slides was observed in 48 samples after 1:1 and 1:4 blood to saline dilution, but only persisted in four samples after washing. Among the DAT+ samples, 57% had agglutination, 87% had spherocytosis, and 45% had both. There was good correlation between spherocytosis and DAT results from the six DAT techniques, but the correlation with autoagglutination was only fair. Clinical follow-up was available for 42 dogs. Of the sample from 12 DAT+ dogs collected during treatment, 10 remained DAT+ when tested 1–24 weeks after initial assessment.

**Conclusions:**

Based upon this comparative prospective survey, all in-clinic and laboratory DAT techniques produced similar results when performed by trained personnel and can therefore be recommended for detection of antibody-coated erythrocytes and immunohematological diagnosis. In addition, use of these tests for monitoring response of IMHA dogs to treatment might be valuable.

**Supplementary Information:**

The online version contains supplementary material available at 10.1186/s40575-021-00107-0.

## Plain English summary

Immune-mediated hemolytic anemia (IMHA) is driven by accelerated destruction of antibody-coated red blood cells (RBCs). The clinical diagnosis of IMHA requires blood tests and can be challenging due to the lack of established reference methods and reagents. We compared routine and specific blood test methods in samples from 126 dogs suspected of having IMHA. In addition, 28 samples from healthy non-anemic dogs served as negative controls. Using a variety of direct antiglobulin (Coombs’) test (DAT) methods, antibody-coated RBCs were detected in approximately half of the samples from dogs with suspected IMHA. The results of four laboratory and two in-clinic DAT kits were comparable and consistent. Marked spherocytosis (small spherical RBCs) and to a lesser extend autoagglutination (clumping of RBCs) also correlated with the DAT results. Furthermore, dogs with DAT positive results were more severely anemic and more likely to have active bone marrow responses compared to dogs with negative DAT results. These concordant results underscore the diagnostic value of both laboratory and in-clinic DAT methods for IMHA in dogs. They confirm marked spherocytosis and a positive DAT result as useful clinical parameters for diagnosing and potentially monitoring this disease.

## Background

Immune-mediated hemolytic anemia (IMHA) is driven by accelerated destruction of antibody-coated red blood cells (RBCs). Approaches to the diagnosis of IMHA in dogs remain controversial, with some investigators and practicing clinicians applying strict hematological and immunological parameters, while others use a combination of RBC agglutination, spherocytosis or responsiveness to immunosuppressive therapy as diagnostic criteria. For many years, we have recommended that IMHA be diagnosed by evidence of in vivo hemolysis and one of three specific immunohematological test results: persistent autoagglutination after three saline washes, marked spherocytosis, and/or a positive DAT result [1–5].

In 2019, the American College of Veterinary Internal Medicine (ACVIM) consensus statement offered similar guidelines, but the data substantiating these recommendations were sparse [6]. There remain disparities regarding the routinely used diagnostic techniques and interpretations of specific test results for diagnosis of IMHA in veterinary clinics as well as in laboratories, as recently illustrated in a small survey by the Veterinary and Comparative Clinical Immunology Society [7].

In human medicine, a positive Coombs’ test result is required for the diagnosis of IMHA [8, 9]. The Coombs’ test was established in 1945 by Robin Coombs, a veterinarian and immunologist at Cambridge, United Kingdom, although Italian scientists reported on the conceptual basis for this test even earlier [10]. The DAT detects bound antibodies (and complement) on the surface of erythrocytes [11, 12]. Species-specific antiglobulins (also known as Coombs’ reagent) are used to detect bound antibodies and/or complement (C3) by an agglutination or other erythrocytic binding reaction. Originally performed as a tube assay, a variety of DAT methods are currently utilized in human diagnostic laboratories, including microtiter plate, capillary, gel minitube, and flow cytometric technologies [8, 13–15]. However, there are no single reference (gold standard) DAT methods or specific antiglobulin reagents established [8, 9, 16], and in-clinic DAT kits are not available in human medicine. Notably, the nature of the antigens to which the autoantibodies bind on the erythrocyte membrane is still not precisely defined [8, 9, 14, 17].

In contrast to human medicine, the DAT is not universally available in canine medicine, and the value of DAT results has been questioned by veterinary clinicians, due to concerns with false negative and false positive DAT results, that are potentially related to ineffective reagents, faulty methods, incorrect test interpretation, and interference by treatments, such as immunosuppressive drugs and transfusions [1–3, 6, 8, 18]. It is the authors’ impression that the DAT is often skipped in the diagnostic approach of IMHA in dogs. Indeed, in a recent study of dogs suspected of having IMHA, only 20% were tested by DAT [19]. As such, a presumptive diagnosis of IMHA is frequently made without evidence of immune-mediated destruction of erythrocytes in anemic dogs [1–3, 7, 20, 21].

The senior author’s (UG) laboratory had introduced and compared several in-clinic and laboratory DAT methods to diagnose IMHA in a small number of dogs in 2014 [3]. In the present study, prior investigations were expanded to include samples from a larger cohort of dogs submitted to a diagnostic laboratory for DAT, and also from healthy control dogs by comparing various, in part novel, means to document erythrocytic immune destruction. In addition to evaluation of marked spherocytosis, saline agglutination test (SAT), and persistent autoagglutination after washing, the presence of antibody-coated erythrocytes was documented by two in-clinic and four laboratory DAT techniques.

## Materials and methods

### Blood samples from dogs

Ethylenediaminetetraacetic acid (EDTA)-anticoagulated blood samples from dogs suspected of having IMHA and for which a DAT was requested were gathered at a major veterinary diagnostic laboratory (Laboklin, GmbH&Co.KG, Bad Kissingen, Germany). Samples with DAT positive (DAT+) or DAT negative (DAT-) results by routine laboratory methods were included, if the left-over sample was ≥ 500 µL and the sample was kept at 4°C for < 6 days prior to investigation. During the study, the routinely performed DAT methods used at the diagnostic laboratory were changed, and, thus, those results were not included. Samples were only included when the veterinarian who performed all tests on each sample (NI), had time to perform these laboratory analyses (requiring about four hours [h]). All veterinarians who submitted samples that were DAT+ results were contacted to obtain information about the dog’s clinical signs in support of a IMHA diagnosis and any available clinical follow-up. They were also offered free follow-up laboratory testing. Despite reminders, clinical information could not be obtained from all dogs. However, for some DAT+ cases, follow-up EDTA blood samples were received one week to six months after the first DAT+ results (initial presentation) to assess response to treatment and resolution of the immune destruction of erythrocytes.

In addition, blood samples from dogs with normal routine blood test results (a complete blood count [CBC] and serum chemistry panel) performed as part of a health screen were selected from samples submitted to the same laboratory (without a request for a routine DAT) and served as negative controls. Some of these samples were also blood typed [22] and used to induce in vitro DAT+ controls by adding *anti-dog erythrocyte antigen (DEA) 4*, *anti-DEA 5*, or *anti-Dal* alloantibodies to coat erythrocytes (see below).

Signalment and routine blood test results (CBC and serum chemistry panel), performed at Laboklin or submitted along with blood samples from the clinics, were gathered from each dog. Only serum samples separated from the cellular components before shipment were included in the bilirubin assessment. The use of left-over blood from the samples routinely submitted for diagnostics at Laboklin was approved by the governmental animal care and committee in Bayern, Germany.

### Laboratory techniques

For each sample, microscopic blood smear examinations, various agglutination tests, and DATs were performed with either whole blood or after centrifugation and washing within 4 h as outlined (Suppl Table S1) according to either manufacturer’s instructions or standard operating procedures. The overnight incubation of the microtiter plate at 4°C required an additional time period (12–15 h).

#### Sample preparation

Initially, each blood sample was visually examined for gross autoagglutination in the submitted EDTA tube and graded semi-quantitatively as 0 (no clumps), 1+ (fine clumps [≤ 1 mm]), or 2+ (granular clumps [> 1 mm]). Tubes were then centrifuged at 240 × g for 5 minutes (min), and after visual inspection, the plasma was separated from packed red blood cells (pRBCs). Plasma appearance was categorized as normal, lipemic, icteric, or weakly to strongly hemolytic from 0 to 3+ : 0 for transparent straw-colored, 1+ for a slight pink coloration, 2+ for a bright red coloration, and 3+ for a dark red discoloration [23].

#### Hemoglobin and packed cell volume

The required amount of EDTA blood and red blood cells (RBCs) for all DATs, agglutination tests, and blood smears was estimated in µL based on the blood’s hemoglobin (Hb) content (HemoCue® Hb 210 + [Brea, CA, USA] or from CBC). When additional EDTA blood was available beyond what was needed for the various tests related to immune destruction of RBCs (see below), the microhematocrit (packed cell volume [PCV]), and plasma Hb (HemoCue®) concentration were measured. When both total blood Hb and PCV values were available, the blood Hb concentration (in g/dL) was multiplied by three to express the predicted hematocrit (Hct) in percent for comparison.

#### Autoagglutination testing before and after washing

Independent of the degree of visual tube autoagglutination, either one or four drops of 0.9% NaCl solution were mixed with one drop of blood to assess autoagglutination macroscopically on separate microscopic slides (1:1 and 1:4 SAT) [3]. Both the microscopic slides and saline were kept at 22°C (room temperature), and blood samples were brought to 22°C prior to testing.

All samples which showed any autoagglutination in the tube and/or on the slide were further tested for persistence of (also referred to as true) autoagglutination after three saline washes [3]. Briefly, one part pRBCs obtained by centrifugation was resuspended in approximately four parts of saline and then centrifuged at 240 × g for 5 min. The supernatant was discarded and the process was repeated twice more. Then one drop of washed pRBCs was mixed on a slide with four drops of saline. The autoagglutination was assessed macroscopically and categorized as present or absent.

#### Microscopic examination of blood smears for spherocytosis, autoagglutination, and ghost cells

Blood smears were prepared 1–5 days after blood collection and were performed along with other tests. Blood smears were fixed and stained (Wright Giemsa stain modified, Sigma-Aldrich, Taufkirchen, Germany) and microscopically examined. Spherocytosis was considered present when ≥ 5 spherocytes were seen in microscopic fields with a 100 × high-power field (hpf) [3, 24, 25]. The presence of ≥ 5 ghost cells/100 × hpf was also noted [17, 26]. Finally, the presence of polychromasia (1+ : 2–7, 2+ : 8–14, 3+ : 15–29, 4+ : > 30 cells/1000 × microscopic monolayer field) and rouleaux formation was assessed on blood smears as present or absent.

#### Direct antiglobulin tests

Erythrocyte-bound antibodies were detected using six DAT techniques with different polyclonal antiglobulin reagents, incubation periods, and temperatures (Suppl Table S1 and Suppl Figure S1). Prior to this prospective laboratory study, protocols for the various DAT techniques were established based upon manufacturers’ instructions and literature, but no formal validation studies were done for any assays. Polyclonal *anti-DEA 4* and *anti-DEA 5* (ABRI, Animal Blood Resources International, Dixon, California) as well as *anti-Dal* (from clinically sensitized dog [22]) alloantibodies were used to induce in vitro positive controls for neutral gel column card (GEL LAB), microcapillary tube (CAPIL), and microtiter plate (MICRO) DAT. *Anti-Dal* was used undiluted, while *anti-DEA 4* and *anti-DEA 5* were diluted 1:8 and 1:2.5, respectively [22].

##### Immunochromatographic strip kit (STRIP KIT) DAT method

The immunochromatographic strip kit DAT (STRIP KIT DAT; Canine Labtest DAT©, Alvedia, Limonest, France) was performed according to the manufacturer’s instructions and as previously described [3]. Since the original description of the STRIP KIT DAT [3], the erythrocytic binding strength at the band, where the antiglobulin (RAD: rabbit anti-dog IgG, IgM, and C3 – canine anti-globulin Coombs’ reagent, MP Biomedicals, Solon, OH, USA) is located, has been enhanced according to the manufacturer. The test results were graded by visual inspection as negative (-) and weakly to strongly positive (1+ to 4+) based on the intensity of the observed antiglobulin test band compared to the strength of the control band. If RBCs stayed at the origin of the strip, it was considered to be due to autoagglutination and thus recorded as a strongly positive result as per manufacturer’s instructions.

##### Gel minitube kit (GEL KIT) DAT method

The gel minitube kit DAT (GEL KIT DAT, Gel Test Canine DAT©, Alvedia) was performed following the manufacturer’s instructions and using a recommended centrifuge with swinging buckets (Hettich EBA 270, Tüttlingen, Germany). While RBC washing is not required, this test was performed twice with a whole blood suspension (5 µL whole blood in 495 µL buffer solution) and with washed RBCs in separate minitubes containing the antiglobulin (RAD) in gel. The test results were graded as negative (-), 1+ , 2+ , 3+ , and 4+ , as described by the manufacturer and previous gel column and tube studies [3, 13, 15, 20]. The neutral gel minitubes (Neutral gel test, Alvedia) were used as additional auto-controls beside the neutral gel column test (see below; both gels lacked incorporated antiglobulins), and results were negative.

##### Laboratory gel column card (GEL LAB) DAT method with neutral gel columns

Each blood sample was tested by the gel column card DAT method with neutral gel columns (GEL LAB DAT) as previously described [3] after RBC washing with saline (negative auto-control) and with one of two different polyclonal canine antiglobulin reagents: GAD (goat anti-dog IgG, IgM, and C3: Canine Coombs Reagent, VMRD, Pullman, WA, USA) or RAD (MP Biomedicals). Briefly, a 1% RBC suspension was prepared by mixing 5 µL pRBCs in 500 µL saline. Then, 13 µL RBC suspension was mixed with 6.3 µL GAD, RAD, or saline (i.e., a 1:2 ratio of antiglobulin solution to RBC suspension) on top of neutral gel columns (NaCl Enzyme Test and Cold Agglutinin ID-card, DiaMed GmbH, Cressier, Switzerland). The gel column cards were incubated for 15 min at 37°C (Incubator IN30, Memmert, Germany) and, thereafter, centrifuged in a special centrifuge (Diamed ID-Centrifuge 6S, DiaMed) for 10 min at 85 × g. The test results were interpreted as for the GEL KIT DAT described above [3, 13]. Care must be taken to avoid the presence of air between the reagent and the red blood cell suspension, as incorrectly mixed solutions might falsify the test result. The problem can be easily solved by gently shaking the sample column until the suspension is mixed and the locked air escapes.

##### Capillary (CAPIL) DAT method

The capillary (CAPIL) DAT was performed as described [3, 13] with both (GAD and RAD) antiglobulins. Briefly, one third of a microcapillary tube (Precision capillary tube 0.4 × 75 mm, Drummond Scientific Company, Broomall, PA, USA) was filled with one of the two antiglobulin reagents, and a 30% washed RBC suspension in saline was added until two thirds of the microcapillary tube was reached. The microcapillary tube was then inverted, incubated in a 60° angle in clay at 22°C for 10 min, and the visual presence (+) or absence (-) of agglutination was then noted. As also observed with the LAB GEL DAT method, air trapped between the blood and the reagent can become an issue by keeping the reagent and the blood apart. This technical issue can be easily solved by gently tapping the microcapillary tube to allow the trapped air to escape [3, 27].

##### Laboratory microtiter plate (MICRO) DAT method

For the microtiter plate (MICRO) DAT method, a 4% RBC suspension was produced by adding 14.4 µL washed pRBCs to 346 µL saline. An 11-step doubling dilution gradient from 1:2 to 1:2048 with each of the two canine antiglobulin reagents described above (GAD [MICRO GAD DAT] and RAD [MICRO RAD DAT]) was set up in a 96-well round bottom microtiter plate (Microtiter plate 96 wells U-Form, Merck, Darmstadt, Germany), as described [3, 28, 29]. To the last well of each horizontal row, 15 µL saline was added as a negative (or autoagglutination) control. The microtiter plate was incubated (Incubator IN30) at three different temperatures: 22°C for 30 min, then 37°C for 30 min, and, finally, at 4°C overnight for 12–15 h. Between each temperature change, the agglutination test result was recorded, and any agglutination was completely dispersed by horizontally shaking of the plate before each re-incubation. Each row was evaluated for the presence (+) or absence (-) of agglutination, and the degree of positivity was based upon the last dilution with observed agglutination from 1:2 to 1:2048 [3]. While all DAT methods were compared to each other, the result presentation focuses on the comparison to the MICRO GAD DAT at 22°C.

##### Laboratory flow cytometric (FLOW) DAT method

The flow cytometric (FLOW) DAT was performed with fluorescein isothiocyanate (FITC)-marked goat anti-dog IgG antibodies (Goat anti-dog IgG [H + L]:FITC, BioRad, Oxfordshire, United Kingdom) and an Attune NxT flow cytometer (Thermo Fisher, Waltham MA, USA) as described [18]. Briefly, 5 µL pRBCs were washed three times with 500 µL phosphate-buffered saline (PBS) containing 0.5% bovine serum albumin (0.5% BSA, FACS Cell Wash, Becton Dickinson GmbH, Heidelberg, Germany, named BSA-PBS) at 200 x g for 15 min. Then, 100 µL of a 1% washed RBC suspension were mixed with 100 µL of the 1:10 diluted antiglobulin reagent and incubated in the dark at 4°C for 30 min. The suspension was washed again three times after incubation and finally resuspended in 200 µL PBS/0.5% BSA before measurement. The scatter plot’s side scatter height (SSC-H) versus forward scatter height (FSC-H), forward scatter width versus (FSC-W) versus FSC-H were applied for gating, and SSC-H versus FITC-H were used to assess the degree of positivity for each sample**.** A washed 1% RBC suspension without antibodies was used as negative control. For each sample, 10,000 events were recorded in a pre-gated region. The sample positivity was stated as percentage of all gated RBCs. All measurements were performed with a FSC wavelength of 240 nm and SSC of 380 nm. Blood samples of 12 clinically healthy dogs were used to establish reference values. Values of > 2 standard deviations (SD) above the mean percentage of positive cells were considered DAT+ .

### Data analyses

The entire dataset of samples submitted for a routine DAT were divided into DAT+ and DAT- groups. Samples were considered DAT+ when they were scored positive by the laboratory MICRO GAD DAT results or scored positive using 4–6 DAT methods (named ≥ 4 DAT+). Samples were considered DAT- when either the MICRO GAD DAT result was negative or fewer than three other DAT method results were positive.

All statistical analyses were performed with SPSS Statistics 25 software [30]. Cohen’s and Fleiss’ kappa [31] (κ)-values and two-sided 95% confidence intervals (95% CI) were calculated to assess the degree of agreement with all possible pairs of DAT methods. κ-value analyses were performed twice: first using samples from dogs submitted for DAT, and thus suspected of having IMHA by the attending veterinarian, and also by including the 28 healthy control dogs, and interpreted with the scale originally established by Landis and Koch [32], with modified category names [33]. A *p*-value of < 0.05 was considered significantly different. The normality assumption was tested with a Shapiro-Wilk test, histograms, box plots and Q-Q diagrams [34]. If not met, a Mann-Whitney U test was performed instead of a t-test [35, 36]. The nominal data were analyzed with chi-squared test or, if not indicated due to small number of samples, a Fisher’s exact test [37, 38]. In addition, the analytical diagnostic sensitivity and specificity were calculated for each method in comparison to the MICRO DAT with GAD at 22°C [39, 40].

## Results

From November 2019 until November 2020, EDTA-anticoagulated blood samples from 126 dogs suspected of having IMHA and for which a routine DAT was requested were analyzed at a major veterinary diagnostic laboratory (Laboklin GmbH&Co.KG, Bad Kissingen, Germany). The blood samples came mainly from Germany but also from a few other European countries. Because of sample limitations (insufficient quantity; too old) and/or operator availability (first author [NI] was not always available to perform extensive testing), an additional 78 DAT+ and 1,033 DAT- samples submitted for routine DAT during the same 13-month period were not included.

### Healthy negative control dogs and positive control samples

Additional EDTA blood samples submitted from a total of 28 healthy dogs were included in this survey. None of them showed anemia, autoagglutination, spherocytosis, or positive DAT results by any of the test methods performed. This DAT- control group was composed of 20 mixed breed and eight purebred dogs with a female to male ratio of 1.55, a median age of nine years, and age range of 1–14 years (Suppl Table S2 and S3, A to C).

In addition, samples from eight of the above 28 negative control dogs were examined after their blood was incubated with polyclonal *anti-DEA 4*, *anti-DEA 5*, or *anti-Dal* to allow coating of RBCs*,* thereby serving as DAT+ control groups. All of these dogs had the common blood type pattern *DEA 4* + *, DEA 5-,* and *Dal*+ [22]. Exposure to *anti*-*DEA 4* and *anti-Dal* induced a positive DAT reaction in all eight dogs with all DAT methods applied, but the degree of positivity differed depending on the dog tested. As expected by their blood type, *anti-DEA 5* did not produce DAT + results in these dogs (Suppl Table S4). Together, these results provide appropriate negative and positive controls which were mostly lacking in previous studies.

### Signalment, blood Hb concentration, and CBC

In the 126 dogs for which a routine DAT was clinically requested, 44 were mixed breed and 78 were purebred dogs with no more than six dogs per breed (breed was not stated for four dogs) and more females than males (ratio 1.41). Neutering status was infrequently mentioned and thus is not reported here. The age ranged between 0.5 to 15 years, with a median of eight years. When comparing the signalment of DAT+ against DAT- dogs, no significant differences were observed, except there were more females in the MICRO GAD DAT+ than DAT- group (Suppl Table S2 and S3 A to C).

Based upon measurements of total blood Hb concentrations and CBC results, 105 of 126 tested dogs were mildly to severely anemic. The DAT+ dogs were more frequently and more severely anemic and more likely to have hyperbilirubinemia than the DAT- dogs (Suppl Table S5 and S6, Suppl Figure S2A, D and S3A, D). More than half of tested dogs showed a reticulocytosis (> 110,000/nL) and 65% of them were DAT+ with the MICRO GAD DAT method and ≥ 4 DAT+ , respectively. The degree of reticulocytosis correlated with microscopically observed polychromasia, but not with the degree of anemia (Suppl Table S5 and S7, Suppl Figure S2C and S3C). The presence of Hb in plasma samples did not differ significantly in DAT+ and DAT- dogs (Suppl Table S5, Suppl Figure S2B and S3B).

### Assessment of blood smear

Ghost cells, as proposed by ACVIM consensus report, were only rarely found and showed a slight association with the ≥ 4 DAT+ group, but not with the MICRO GAD DAT+ results (Table 1). Marked spherocytosis was seen frequently and significantly more often in DAT+ than DAT- dogs (Table 1). Only one of 58 dogs with spherocytosis was DAT- by all methods. The presence of rouleaux formation did not differ significantly between the DAT+ and DAT- groups and was not associated with the presence of agglutination (Table 1).

**Table 1:** Spherocytosis, ghost cells, rouleaux, and macroscopic agglutination test results compared to DAT results in 126 dogs suspected to have IMHA

Test group	DAT result	Spherocytosis^a^		Ghost cells^a^			Rouleaux^a^			SAT 1:1		SAT 1:4		Persistent agglutination	
		Yes	No	Yes		No	Yes	No		Yes	No	Yes	No	Yes	No
**MICRO DAT+ **	Yes	54	8	10		52	13	49		38	29	38	29	4	63
	No	4	54	3		55	13	45		10	49	10	49	0	59
	*p value*	** < *.001***		*.077*			*1.0*			** < *.001***		** < *.001***		*.122*	
** ≥ 4 DAT+ **	Yes	53	9	11		51	14	48		39	28	39	28	4	63
	No	5	53	2		56	12	46		9	50	9	50	0	59
	*p value*	** < *.001***		***.017***			*0.828*			** < *.001***		** < *.001***		*.122*	

Groups were compared by chi-squared test

*SAT* saline agglutination test, *DAT* direct antiglobulin test, *MICRO DAT* microtiter plate DAT with goat anti-dog IgG, IgM, and C3 reagent incubated at 22°C*,* ≥ *4 DAT*+ at least four different direct antiglobulin test results were positive among the five to six performed DATs

^a^Blood smears from six dogs could not be assessed due to strong agglutination, except for the presence of autoagglutination

### Evaluation for macroscopic autoagglutination

**Fig. 1:**
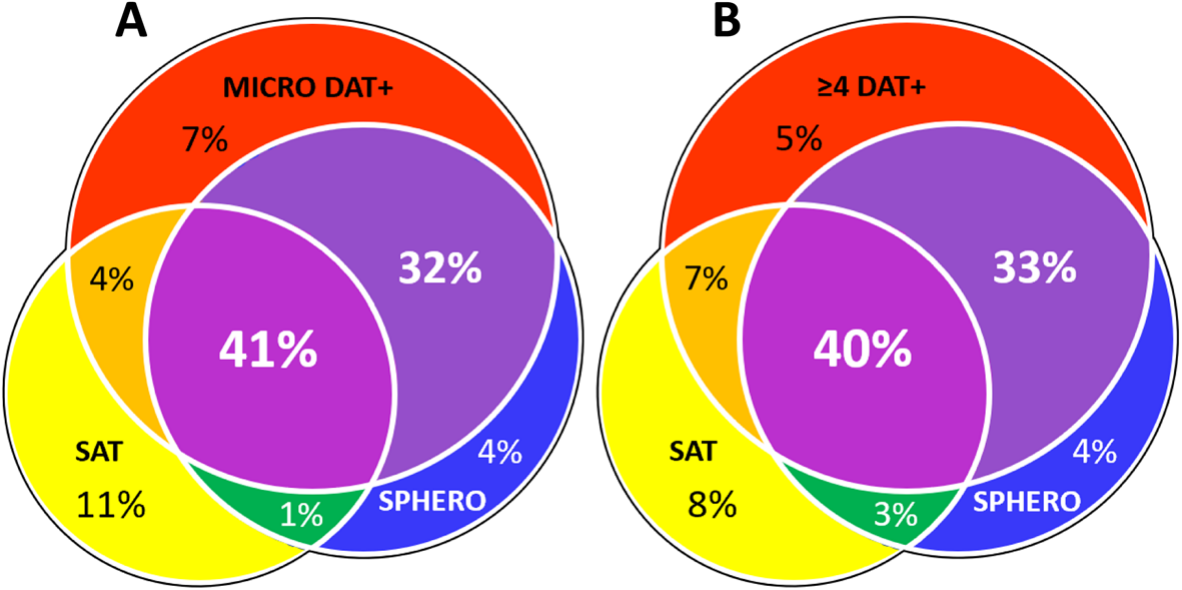
Venn diagrams comparing agglutination, spherocytosis, and DAT+ results in 120 dogs suspected to have IMHA. Microtiter plate direct antiglobulin test (MICRO) results with goat anti-canine IgG, IgM, and C3 incubated at 22°C (**A**) and ≥ 4 DAT+ results (**B**). Six of 126 cases were excluded due to strong agglutination making the evaluation of the presence of spherocytosis impossible. MICRO DAT+ : microtiter plate direct antiglobulin test method with goat anti-dog IgG, IgM, and C3 incubated at 22°C; SAT: 1:1 macroscopic saline agglutination test before washing; SPHERO: spherocytosis defined as ≥ 5 spherocytes per 100 high power field

Autoagglutination of EDTA blood in tubes was visually observed more often in samples from DAT+ dogs than from DAT- dogs (Table 1). All samples with visual tube agglutination also showed macroscopic agglutination by SAT in undiluted blood or after 1:1 or 1:4 saline dilution. Ten additional samples showed macroscopic autoagglutination in SAT without tube agglutination (Table 1). Agglutination was noted in 48 samples with ~ 30% having a granular pattern, and the remainder exhibiting only fine agglutination. Finally, after washing blood three times with saline, only four samples were still slightly agglutinated (Table 1). Therefore, despite originally agglutinating, analysis of DAT could be attempted in all dogs and in nearly all cases was interpretable (see below). Remarkably, DAT results were negative in ten of the agglutinating samples (~ 20%) (Fig. 1).

### Direct antiglobulin test (DAT) results

#### Laboratory microtiter plate (MICRO) DAT method

**Fig. 2:**
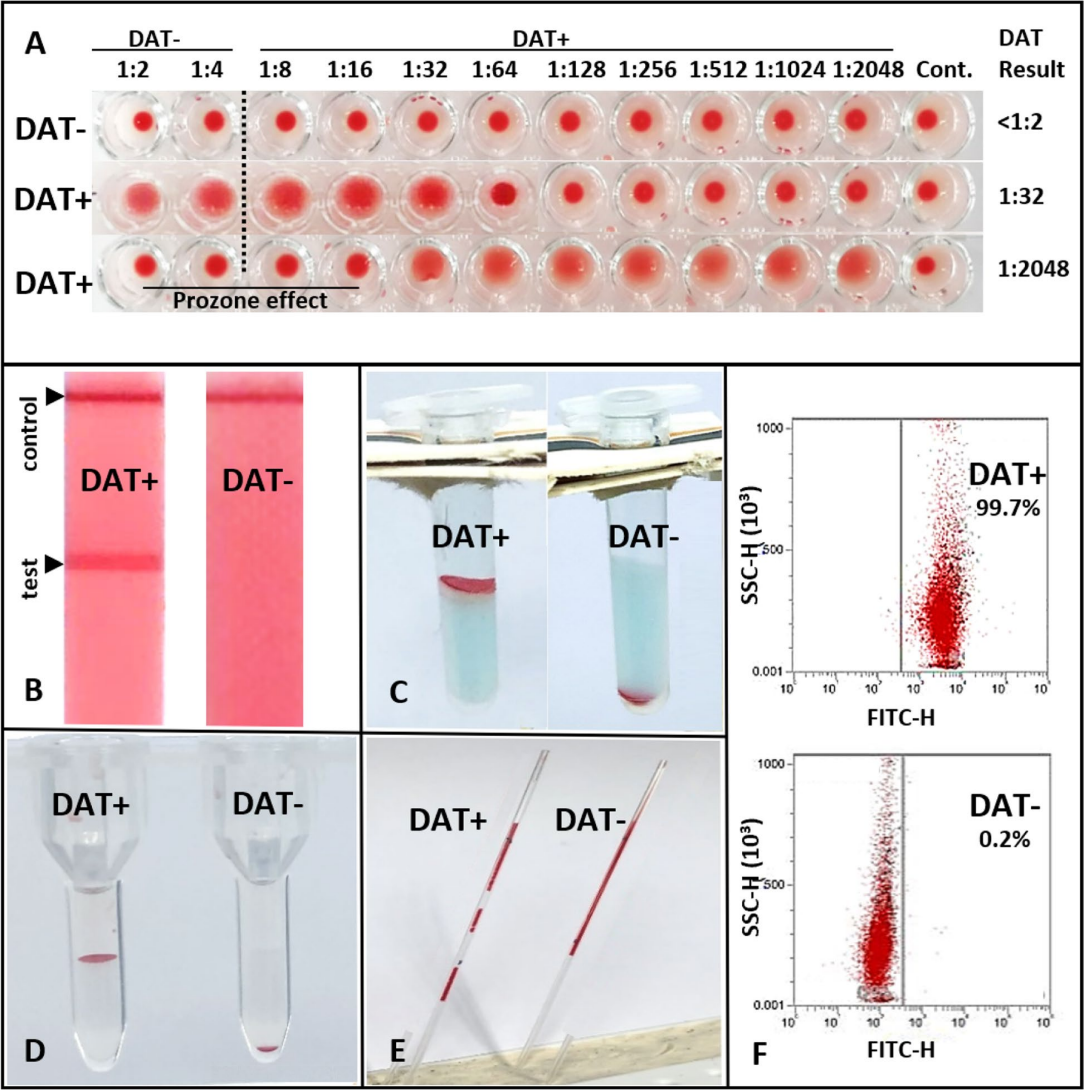
Representative positive and negative direct antiglobulin test (DAT) results performed with different methods (**A**) microtiter plate; **B** in-clinic immunochromatographic strip kit; **C** in-clinic gel minitube kit; **D** neutral gel column card with added antiglobulin; **E** microcapillary tube; and **E** flow cytometry DAT± : direct antiglobulin test positive/negative result; Cont: control (saline but no antiglobulin added)

MICRO DAT analysis using GAD at 22°C, identified 67 of 126 samples as positive, with strongly to moderately and rarely weakly positive DAT titers (Table 2). In half of the MICRO GAD DAT+ samples, a prozone effect was observed in moderately to strongly DAT+ samples (titer ≥ 1:256 with MICRO GAD 22°C, Table 2 and Fig. 2A). Among the 59 MICRO GAD DAT- dogs, there were 18 samples that tested DA + by one (*n* = 15) and rarely more (1 DAT+  = 15, 2 DAT+  = 2, 3 DAT+  = 1) of the other DAT methods, and 16 of them were only weakly DAT+ (Table 3).

**Table 2:** Direct antiglobulin test results compared to titers and prozone effect in dogs suspected to have IMHA

MICRO DAT	Titer	Frequency n (%)	Prozone n (%)	≥ 4 DAT+ n (%)
**DAT-**	< 1:2	56 (44)	0	3 (4)
	1:2	1 (1)	0	1 (1)
	1:4	2 (2)	0	2 (3)
**DAT + **	1:32	2 (20	0	1 (1)
	1:64	1 (1)	0	1 (1)
	1:256	4 (3)	1 (3)	4 (6)
	1:512	6 (5)	3 (9)	5 (7)
	1:1024	6 (5)	0	5 (7)
	1:2048	48 (38)	30 (88)	45 (67)
**Total Dogs n**		**126**	**34**	**67**

Titers of 1:8 and 1:16 were not seen; n: number of dogs

*DAT* direct antiglobulin test, *MICRO DAT* microtiter plate DAT with goat anti-dog IgG, IgM, and C3 reagent incubated at 22°C, ≥ *4 DAT*+ at least four different DAT results were positive among the five to six performed DATs

**Table 3:** Direct antiglobulin test results with five methods in dogs suspected to have IMHA

DAT Method	DAT-	1 DAT+	2 DAT+	3 DAT+	4 DAT+	5 DAT+
MICRO	35	0	1	6	13	53
GEL KIT	35	2	1	4	14	53
GEL LAB	35	7	0	1	5	53
STRIP KIT	35	6	2	5	13	53
CAPIL	35	0	0	5	11	53
**Total** (*n* = 126)	**35**	**15**	**2**	**7**	**14**	**53**

Results with MICRO DAT include all reagents and temperature; results of GEL LAB and CAPIL DAT with both reagents. Any positivity with one or more reagents or temperatures was considered DAT+

*DAT* Direct antiglobulin test, *STRIP KIT* in-clinic immunochromatographic strip kit, *CAPIL* microcapillary tube, *MICRO* microtiter plate, *GEL KIT* in-clinic gel minitube kit, *GEL LAB* neutral gel column card with added antiglobulin, FLOW was excluded because of lower number of samples tested

Performing the MICRO GAD DAT at 37°C and 4°C produced very similar results to those obtained at 22°C (Suppl Table S8). Likewise, replacement of GAD with the RAD antiglobulin and evaluation of the DAT at each temperature revealed similar results (Suppl Table S8).

#### Immunochromatographic strip kit (STRIP KIT) DAT method

Using the STRIP KIT DAT method with RAD antiglobulin at 22°C (Alvedia), 79 of 126 washed blood samples tested DAT+ (Suppl Table S9 and Fig. 2B). This included seven samples showing a strong band at the origin (bottom) of the strip, indicative of autoagglutination. These samples were also agglutinating on slides (and two of them showed persistent agglutination), tested DAT+ by other methods, and were considered strongly DAT+ . Strongly positive (4+ and 3+) bands were seen with the STRIP KIT DAT in 30 dogs, with half of the remaining DAT+ samples showing moderate (2+) or weak (1+) binding at the strip’s test site, respectively (Suppl Table S9). All samples testing DAT+ by MICRO GAD DAT were STRIP KIT DAT+ , except for one, and were frequently also DAT+ by other DAT methods. In addition, 11 MICRO DAT- samples showed moderately (*n* = 3) to weakly (*n* = 8) positive band strength by STRIP KIT DAT (Suppl Table S9).

#### Gel minitube kit (GEL KIT) DAT method

Using unwashed blood with the GEL KIT DAT and RAD at 22°C (Alvedia), 74 of 126 samples were identified as DAT+ . For all GEL KIT DAT+ samples the RBCs accumulated at the top (4+ or 3+) allowing for easy test interpretation (Fig. 2C). However, a double population of RBCs at the top and bottom of gel minitubes was noted in 13 samples that were still graded as 4+ (Suppl Table S9). All samples testing DAT+ by routine MICRO GAD DAT were DAT+ by the GEL KIT DAT, except for three (those were, however, GEL LAB DAT+). All samples with ≥ 4 DAT+ results were also GEL KIT DAT+ . Based upon the clinicopathological information received for 42 cases, only one of these dogs was transfused before testing. Washing the RBCs prior to performing the GEL KIT DAT produced similar results (Suppl Table S10).

#### Laboratory gel card (GEL LAB) DAT method with neutral gel column card

Utilizing the GEL LAB DAT method with GAD antiglobulin and neutral gel column cards, 52 of 126 samples were DAT+ . The degree of agglutination reaction was strong (4+ or 3+ , Fig. 2D) for 38 samples, and the DAT results were moderately (2+) or weakly (1+) positive for an additional four and ten samples, respectively. Except for five cases, all GEL LAB GAD DAT+ samples were also positive with MICRO GAD DAT and most other DAT methods (except for two). In addition, 20 samples were GEL LAB DAT- but MICRO GAD DAT+ (Suppl Table S9), and 17 of these 20 cases were DAT+ by all other DAT methods.

Replacing the GAD with the RAD antiglobulin reagent produced similar GEL LAB DAT results. The degree of agglutination was strong (4+ or 3+) for 42 samples, and moderately (2+) or weakly (1+) positive for seven and 12 samples, respectively. All GEL LAB RAD DAT+ samples were also positive with MICRO GAD DAT except for seven weakly and four strongly GEL LAB DAT+ samples (Suppl Table S9). Furthermore, 17 GEL LAB DAT- samples with RAD were MICRO GAD DAT+ ; and similarly, 14 of these 17 samples were DAT+ with all other DAT methods.

#### Capillary (CAPIL) DAT method

The capillary DAT utilizing a 30% washed RBC suspension and either the GAD or RAD reagent, revealed that 55 and 68 of 126 tested samples were DAT+ , respectively (Suppl Table S9 and Fig. 2E). All CAPIL DAT+ samples were also positive by the MICRO GAD DAT (except for four and six cases for GAD and RAD, respectively) and by most other DAT methods (≥ 4 DAT+ ; except for one and five cases, respectively). A discordance of CAPIL DAT- and MICRO GAD DAT+ was rarely observed with RAD (five cases) but more frequently when GAD was used as the antiglobulin (16 cases) in the CAPIL DAT method (Suppl Table S9).

#### Laboratory flow cytometric (FLOW) DAT method

**Fig. 3:**
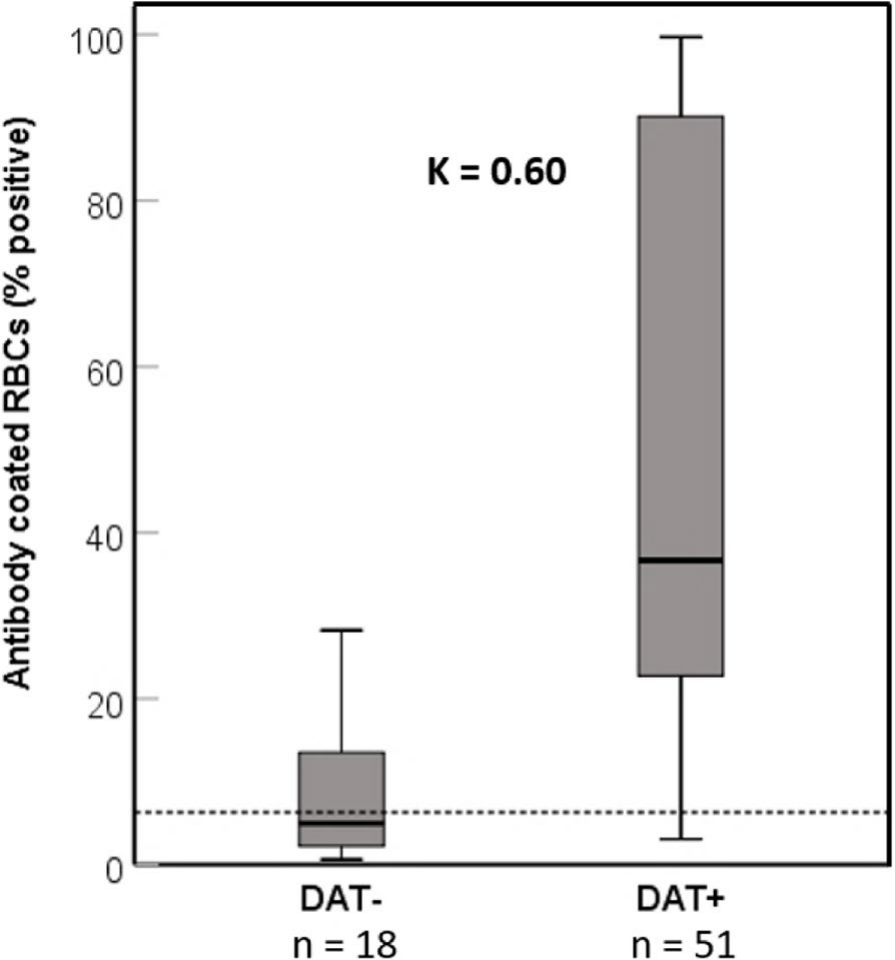
Comparison of flow cytometric and microtiter plate DAT results in 69 dogs suspected to have IMHA. DAT: direct antiglobulin test. Red blood cells (RBCs) marked positive with FITC goat anti-dog IgG were expressed in percentages. The dotted line represents upper limit of normal (6%; mean + 2 SD of 13 control samples). Groups were compared by Cohen’s kappa, κ = .60 (95% CI, .41—.80, *p* < .001). Interpretation of κ values: very good agreement (≥ .81); good agreement (≥ 0.61); moderate agreement (≥ .41); fair agreement (≥ .21), as per Landis and Koch [30] and adapted by Brennan and Silman [31]; SD: standard deviation

The setup of the flow cytometric DAT method was initially hampered by interfering RBC agglutination leading to loss of RBCs and technical flow problems and thus was only included for the second half of the samples. Utilizing an anti-IgG specific antiglobulin reagent, the proportion of goat anti-dog FITC marked IgG antiglobulin-bound RBCs observed among the 69 flow cytometrically tested samples ranged from 1 to 100%. With the established < 6% positive erythrocytes as unspecific background, based on our negative control dogs, and thus categorized as DAT-, 51 samples were FLOW DAT+ . The amount of bound antiglobulin varied from 8 to 100% for DAT+ samples (Figs. 2F and 3). Of the 51 FLOW DAT+ samples, 41 were also MICRO GAD DAT+ . Two FLOW DAT- samples were in the group of ≥ 4 DAT+ , and five FLOW DAT+ samples were DAT- by all other methods (Suppl Table S9).

### Comparative analyses of immunohematological test results

The Cohen κ-values of all possible pairs of DAT methods and their confidence interval (CI) are represented for the 126 dogs suspected of having IMHA in Table 4 and Suppl Table S11. The Fleiss kappa value overall agreement was 0.73 (CI 0.70-0.75, *p* < 0.001) for all 126 samples for which a DAT was requested and 0.79 (CI 0.77-0.81, *p* < 0.001) when including the 28 samples from healthy control dogs, thus both reflect a “good agreement” [31]. In addition, the Cohen’s κ-values were analyzed for all dogs including the 28 healthy control dogs in Suppl Table S12. The MICRO DAT with GAD at 22°C results showed a very good agreement (κ = 0.81–1.0) with each reagent and temperature. The results of CAPIL DAT with RAD also showed a very good agreement with MICRO DAT and with the GEL KIT DAT results. The CAPIL DAT with GAD, the STRIP KIT DAT, the FLOW DAT, and the GEL LAB DAT with GAD revealed good agreement when compared to every other DAT method (κ = 0.61-0.8), while a comparison of the GEL LAB DAT with RAD showed a moderate agreement (κ = 0.41-0.6) [32].

When comparing the results of the MICRO DAT with GAD at 22°C as a reference ("gold standard") method, all other DAT methods showed an analytical sensitivity > 90%, except for the CAPIL DAT with GAD and the GEL LAB DAT with either antiglobulins. The analytical specificity of DAT results was also > 90%, except for the STRIP KIT DAT, FLOW DAT, and GEL LAB DAT with RAD before washing (Table 5).

**Table 4:**
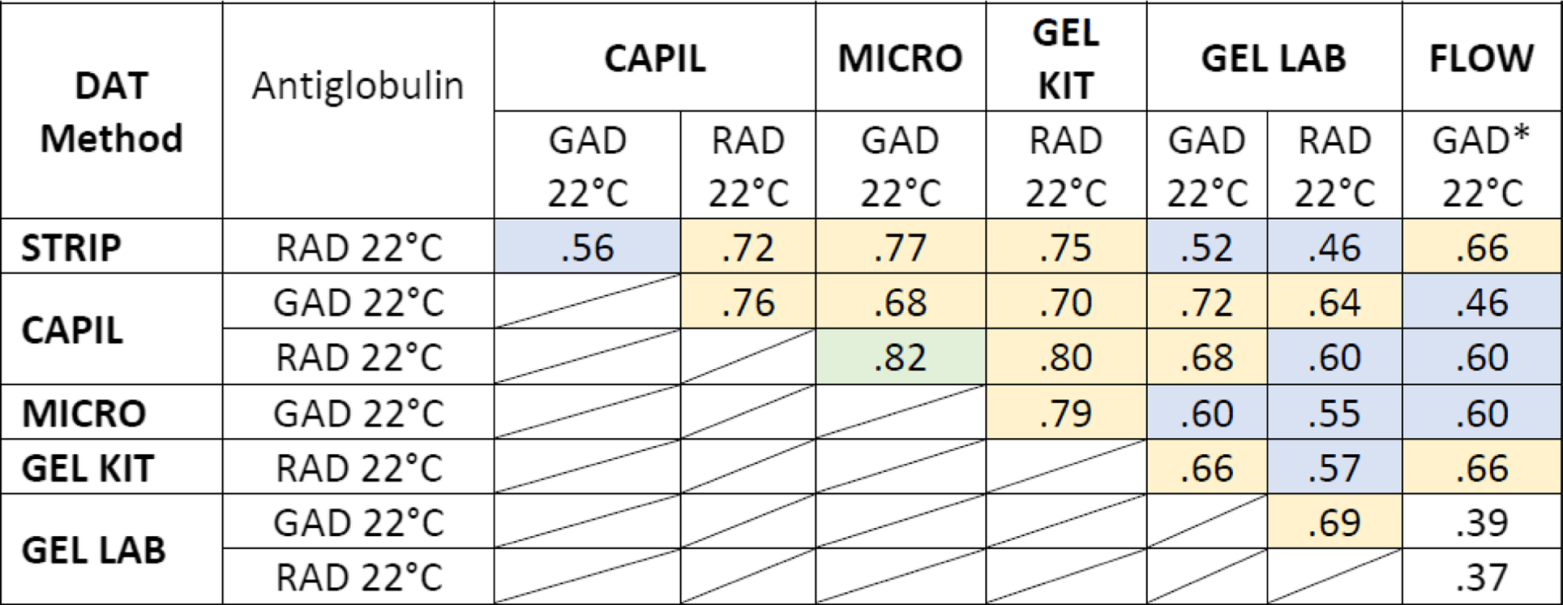
Comparison of Cohen’s kappa (κ) values of six direct antiglobulin test (DAT) results from 126 dogs suspected to have IMHA

DAT: direct antiglobulin test, **DAT methods**: STRIP KIT: in-clinic immunochromatographic strip kit; FLOW: flow cytometry (*n* = 69); CAPIL: microcapillary tube; MICRO: microtiter plate; GEL KIT: in-clinic gel minitube kit; GEL LAB: neutral gel column card with added antiglobulin **Reagents**: GAD: goat anti-dog IgG, IgM, and C3; RAD: rabbit anti-dog IgG, IgM, and C3, GAD*: FITC-marked goat anti-dog IgG (H + L) **Interpretation of κ values**: Groups were compared by Cohen’s kappa, κ = .37 to .82, *p* < .001 Green: very good agreement (≥ .81); yellow: good agreement (≥ .61); blue: moderate agreement (≥ .41); white: fair agreement (≥ .21), as per Landis and Koch [32] and adapted by Brennan and Silman [33]

**Table 5:**
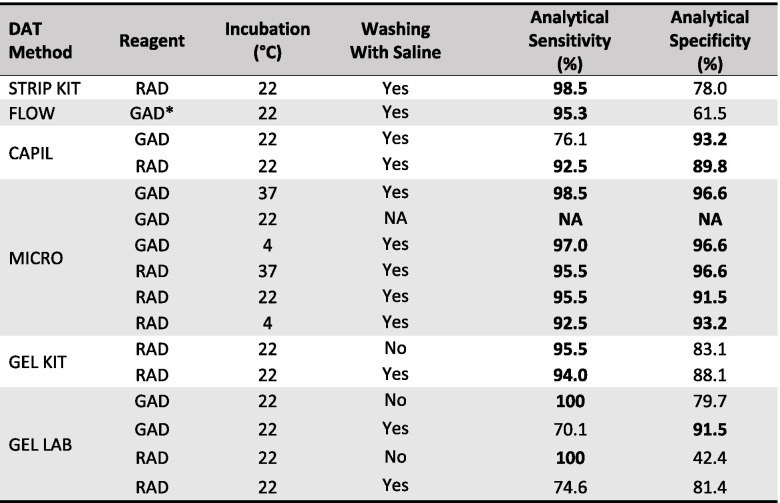
Results of six DAT methods compared to microtiter plate DAT in 126 dogs suspected to have IMHA

*DAT:* Direct antiglobulin test

**DAT methods:** STRIP: in-clinic immunochromatographic strip kit; FLOW: flow cytometry (*n* = 69); CAPIL: microcapillary tube; MICRO: microtiter plate; GEL KIT: in-clinic gel minitube kit; GEL LAB: neutral gel column card with added antiglobulin

**Reagents:** GAD: goat anti-dog IgG, IgM, and C3; RAD: rabbit anti-dog IgG, IgM, and C3, GAD*: FITC-marked goat anti-dog IgG (H + L)

**Interpretation:** > 90% (bold) represent excellent analytical sensitivity and specificity values

NA: not applicable, because everything was compared to MICRO GAD 22°C

The Cohen κ-values, analytical specificity, and sensitivity of the different agglutination tests were modest with a κ-value ranging from 0.39 before to 0.05 after washing (Suppl Table S13). The sensitivity for agglutination ranged from 6% before washing to 57% after washing, and the specificity from 83% before washing to 100% after washing when compared to the MICRO GAD DAT at 22°C. The analytical sensitivity for spherocytosis was 87% and the specificity 93%.

### Follow-up test results for 12 DAT+ dogs

All 67 dogs with DAT+ results were followed up by contacting the submitting clinic (including reminders) with the recommendation that they retest during treatment. In a total of 42 responses, 13 DAT+ dogs were reported to have IMHA and died or were euthanized. The other 29 DAT+ dogs were also clinically diagnosed with IMHA, but were either clinically stable (*n* = 16) or recovered (*n* = 13).

Despite the encouragement for retesting, samples for immunohematological reassessment were only received from 12 of the DAT+ dogs (twice for three dogs) at different time intervals from diagnosis. Seven still showed clinical signs and five were clinically assessed as stable at the time of testing. After 1–2 weeks, the tested dogs were all still anemic (*n* = 4). From week 4 post-treatment, all dogs retested were no longer anemic, except for one, which was still anemic at week 24. Only three dogs were DAT-, while all others (*n* = 9) remained DAT+ when retested between 1 and 24 weeks post-diagnosis. Those dogs which became DAT- also resolved their spherocytosis and autoagglutination.

Furthermore, three DAT+ dogs tested at weeks 2, 8, and 12 post-treatment were reassessed at weeks 19, 20, and 24, respectively. Two of the samples were still DAT+ and had spherocytosis and hemolysis, and one was still macroscopically agglutinating at first retesting (Table 6). At the second retesting, two dogs were DAT-, and had no spherocytosis, and the other still was anemic and had a DAT+ result. That dog was euthanized shortly thereafter.

**Table 6:** Routine blood and immunohematological test results of 12 DAT + dogs with IMHA at time of original testing and during follow-up

Dog #	Weeks from diagnosis	**Hematology**					**DAT**				
		**PCV** **(%)**	**Free Hb (g/dL)**	**Reticulo-cytosis**	**Aggluti-nation**	**Sphero-cytosis**	**STRIP KIT**	**GEL KIT**	**GEL LAB**	**MICRO**	**CAPIL**
1	1	15/26	4.9/0.6	NA/ +	- / -	+ / +	3+/2+	4+ /4+	4+/3+	1:2048/1:2048	+ / +
2	1	21/24	NA/0.0	+ / +	- / -	+ / +	1+/ -	4+/ -	2+/ -	1:2048/ -	- / -
3	2/19	26/36/40	0.1/0.1/NA	+ / + / +	+ / - / -	+ / - / -	4+ / - / -	4+/ -	4+/ -	1:128/ -/ -	- /-/ -
4	2	37/35	1.6/1.2	- /NA	+ / -	+ / +	1+/ -	4+/4+	4+/4+	1:2048/ -	+ / -
5	4	24/40	6.0/0.7	NA/ +	- / -	+ / -	3+/1+	4+/ -	4+/4+	1:2048/1:2048	+ / -
6	4	27/37	0.2/0.2	NA/ -	+ / +	+ / -	3+/2+	4+/4+	4+/ -*	1:512/ -	- / -
7	6	16/58	0.1/0.3	+ / -	- / -	- / -	4+/ -	4+/ -	4+/ -	1:32/ -	+ / -
8	7	13/42	2.3/2.6	+ / +	+ / +	+ / +	4+/1+	4+/3+	4+/ -*	1:128/1:2048	+ / +
9	8/20	26/42/47	3.1/0.3/1.6	+ / + / -	- /-/ -	+ / - / +	2+/3+/2+	4+/4+/4+	4+/4+/2+	1:2048/1:2048/1:2048	+ / + / -*
10	10	18/53	0.4/0.1	- / -	+ / -	+ / -	2+/3+	4+/ -	4+/3+	1:2048/1:2048	+ / -
11	12/24	7/40/10	0.4/0.1/1.0	+ / - /NA	+ / - / +	+ /- / +	4+/1+/2+	4+/4+/4+	4+/2+/4+	1:2048/1:2048/1:2048	+/-* / +
12	14	33/37	0.1/0.1	+ / -	- / -	- / -	4+/3+	3+/ -	1+/ -	1:2/ -	+ / -

A few cases were tested two times during therapy, and thus two or three values are shown

The values prior, and during or after therapy are separated by separated by ‘/’

STRIP KIT: in-clinic immunochromatographic strip kit (DAT+ graded from 1+ to 4+), GEL KIT: in-clinic gel minitube kit (DAT+ graded from 1+ to 4+), GEL LAB: neutral gel column card with added antiglobulin (DAT+ graded from 1+ to 4+), MICRO: microtiter plate (titers equal or below to 1:4 were considered DAT-), CAPIL: microcapillary tube, Spherocytosis defined as ≥ 5 spherocytes per 100 × objective

GEL LAB, MICRO and CAPIL tested with rabbit anti-dog IgG, IgM, and C3 (RAD) and goat anti-dog IgG, IgM, and C3 (GAD) reagents

NA: not available, - : negative, + : positive, *: positive with RAD

## Discussion

While reports of IMHA in dogs are widespread, the diagnostic approach of the disease remains challenging and controversial, and rarely leads to a definitive diagnosis [2, 4, 25, 41]. IMHA is driven by antibody-mediated destruction of RBCs, however no reference (gold standard) diagnostic DAT method has been established so far, and no preferred antiglobulin has been identified yet [3, 6, 8, 25, 42]. To determine the variability and accuracy of currently used methods, we undertook a prospective comparative study of immunohematological tests for canine IMHA. In this large cohort of 126 dogs suspected of having IMHA and 28 healthy control dogs about half of the samples tested had evidence of antibody-coated erythrocytes regardless of what immunohematological test was used. Moreover, we obtained similar and consistent results using various DAT methods. Our results underscore the usefulness of various DAT methods in the diagnosis of IMHA.

### Signalment and routine blood test results of DAT+ and DAT- dogs

With respect to signalment, middle-aged and female dogs were overrepresented as reported in prior retrospective surveys [43–45], but there was neither an apparent breed predilection among dogs for which a DAT was requested nor for those with DAT+ results. In past studies the Cocker Spaniel was overrepresented [24, 43, 45–48], and a recent survey from the United Kingdom suggested a few other breeds with higher odd ratios for non-regenerative IMHA [19].

Among the samples tested in this study, dogs with a positive DAT result had more frequent and more severe anemia (Hct < 18%) as was previously described [44, 46], and the anemia was more likely regenerative in contrast to those in the DAT- group [19, 25, 43]. While there is no published study to directly compare our observations to, it is likely that some DAT- dogs had non-hemolytic anemias. In this study, both DAT+ and DAT- samples appeared hemolyzed, but the degree of hemolysis was higher in DAT+ samples, which may represent an artifact caused by in vitro lysis during shipment and storage (up to 5 days from collection). It is therefore not surprising to find discrepancies between Hb, PCV, and Hct values observed in the study reported here and in clinical practice [49]. When serum chemistry was available, we found hyperbilirubinemia more frequently in DAT+ dogs as expected in dogs with hemolytic anemia [46].

Similar to prior surveys of dogs with IMHA [25, 43, 44], not all DAT+ dogs showed evidence of erythroid regeneration. This may be due to pre-regenerative anemia (first 2–3 days of anemia), mild anemia, or inflammatory and necrotic processes associated with IMHA in dogs, leading to inhibition of adequate erythroid regeneration in the bone marrow [19, 50, 51]. Varied erythroid responses and bone marrow cytological and histological abnormalities have been documented in dogs with IMHA, but time-course was not evaluated [19, 51, 52].

### Spherocytosis

In the present study, ≥ 5 spherocytes per high power microscopic field [6, 24, 25] were primarily seen in DAT+ dogs, with only four exceptions. Low numbers of spherocytes may be observed in many disease processes [1, 53]. In addition, hereditary spherocytosis, although rare in dogs, is associated with severe spherocytosis [54, 55]. Moreover, spherocytes may also represent artifacts and are commonly seen in thicker areas of blood smears [56]. Thus, in dogs with spherocytosis, DAT testing can play an important role in the diagnostic process, with DAT- results helping to rule out IMHA. In addition, although spherocytosis provides strong evidence for IMHA, DAT+ dogs without spherocytosis are not uncommon, representing 5 to 25% in previous studies [56], which correlates well with our finding of 13% of the DAT+ animals being negative for spherocytosis.

### Autoagglutination and SAT

There is considerable controversy regarding the best means to assess and interpret autoagglutination in dogs, and when noted, whether a DAT could and should still be performed [3, 4, 6, 20]. As expected, more DAT+ samples agglutinated than did DAT- samples in the present study. In fact, in many blood samples agglutination occurred in the EDTA tube, and persisted after 1:1 and 1:4 saline dilution. Previous studies noted that 42–87% of samples from dogs clinically suspected to have IMHA showed positive slide/saline agglutination [43, 44, 48]. In the current study, 48% showed positive SAT results, but one fifth of macroscopically agglutinating samples were DAT-. The proportion of agglutinating DAT- samples is also similar to prior smaller studies [3, 24, 46]. Thus, macroscopic agglutination in the tube and/or a positive SAT result do not document antibody-bound erythrocytes and are not truly diagnostic for IMHA.

While the SAT is recommended by the ACVIM consensus statement [6], we had questioned its value in the past [1, 5]. A recent study also suggested that a SAT using a 1:1 and 1:4 blood to phosphate-buffered saline dilution with microscopic evaluation was not a useful diagnostic test for IMHA in dogs [20]. However, the diagnostic accuracy of a 1:49 dilution SAT with microscopic evaluation was much higher, which was attributed to a reduction in non-specific agglutination as happens with saline washing of RBCs [20]. We have previously advocated three saline washes to remove all potentially interfering plasma components and EDTA, as it is standard in human immunohematology [8]. Indeed only four of 48 samples still revealed minor macroscopic agglutination after washing in the current study. These findings suggest that three saline washes should be seen as the standard procedure for immunodiagnostics in dogs [57]. While it is possible that unknown low affinity autoantibodies could dissociate from the RBC surface in the presence of saline [58], such low-affinity antibodies are rarely documented in humans and have not been identified in dogs. Finally, results of agglutination after washing with saline are rarely reported in dogs [20, 25, 43, 59].

Rouleaux formation did not appear to cause the observed macroscopic autoagglutination, as a combination of agglutination and rouleaux were noted in only a few (8%) cases in the present study. It should also be noted that rouleaux only cause microscopic and not macroscopic agglutination [56].

### Direct antiglobulin test (DAT)

As there is skepticism concerning the value of the DAT in canine medicine, due in part to the lack of a reference (gold standard) for any DAT technique and antiglobulin selection [25, 60, 61], we undertook this extensive comparative analysis of multiple techniques currently in use in clinics and laboratories. Our results demonstrate encouragingly good concordance between all DAT methods applied. Because making a definitive diagnosis of IMHA is challenging and immunodiagnostic methods applied vary and are not standardized, accurate assessments of specificity and sensitivity of the various tests are difficult to carry out in the field. Thus, our comparative study of six DAT methods in a controlled laboratory setting provides reassuring evidence that the results of all DAT methods are in overall good agreement, as discussed further below.

The microtiter plate (MICRO) DAT method had been extensively used in human and veterinary laboratories [3, 8, 13–15, 42, 59, 62]. We applied it here as the reference (gold standard) method and demonstrated very good analytical sensitivity and specificity (ranging between 92–99%), even at different temperatures and with two different polyclonal reagents. Thus, the simple room temperature condition appears to be sufficient. Prior studies [18, 21, 59, 63] show similar specificity, from 95 to 100%, but lower sensitivity, between 53 and 82%, for IMHA. However, sensitivity in those studies were based on a clinical diagnosis of IMHA, and not necessarily on documentation of immune destruction of erythrocytes.

The results of the in-clinic immunochromatographic strip kit (STRIP KIT) DAT test with RAD at 22°C in the present study concurs with a prior smaller DAT study regarding analytic sensitivity, but appeared to be slightly less specific [3]. The STRIP KIT test is also used for *DEA 1* blood typing, where its specificity is described to be very high [64]*.* A limitation of this method appears to be the rare occurrence (5%) of autoagglutination at the bottom of the strip, thereby impeding the normal flow of erythrocytes past the test and control bands. This occurred in 7 of our 154 samples which all showed macroscopic agglutination. Moreover, the interpretation of weakly positive DAT reactions can be problematic in clinical practice, but occurred less commonly than in our prior limited study [3].

While washing the cells is recommended prior to most DAT methods, this study demonstrates that the in-clinic gel minitube kit (GEL KIT) DAT results with and without washing are almost identical. Thus, samples for the gel test do not necessarily need to be washed, as long as the negative gel control microtube is negative. Due to its recent introduction, this method has never before been studied in comparison to other DAT techniques and has only been compared to SAT in one previous study [20]. Notably, our results identify the GEL KIT as the quickest and most reliable in-clinic DAT, suggesting that it represents an invaluable tool for differential diagnosis of IMHA in clinical practice.

The neutral gel column card (GEL LAB) DAT is often used as a standard test in human medicine, where its ease of result interpretation and high sensitivity have been praised [15, 65]. The first dog-specific commercial laboratory gel DAT was tested and recommended in two previous publications [3, 62]. Studies comparing neutral gel columns to microtiter plates reported moderate to very good agreement [32, 33]). One laboratory [62] reported agreement slightly lower than the values reported in the current study. Another study [3] describes a very good agreement with the MICRO DAT method. Since that original canine gel column product was discontinued shortly after its introduction, we used neutral gel cards and added a canine-specific antiglobulin reagent in the reaction chamber. This assay was easy to perform and produced very similar results with either GAD or RAD, comparable to the other DAT methods.

Similar to our prior smaller study using the microcapillary tube (CAPIL) DAT [3], 85% MICRO DAT+ samples were CAPIL DAT+ , with an analytical sensitivity and specificity above 90% achieved. The CAPIL DAT is frequently performed in human medicine, particularly in blood banking laboratories [13] to assess blood type incompatibilities. Notably, the simplicity of this technique could make it attractive as an in-clinic and laboratory screening test, when more advanced DAT methods and DAT kits are not available.

While our study data for flow cytometry (FLOW) DAT only tested 69 of 123 dogs, the results were similar to other DAT results. However, there was a somewhat larger proportion of discordant DAT+ and DAT- results when compared with other DAT methods, revealing a very good sensitivity (95%), but a low specificity (62%). The percentage of positive also lacked any specific association with the degree of anemia and hemolysis. Prior studies of FLOW DAT are limited, but reveal sensitivities and specificities ranging from 74 to 100%, when compared to a clinical diagnosis of IMHA [18, 21, 60, 63]. These variations may be related to different standard methods used. Prior limited studies with FLOW DAT suggested that the number of antibody positive RBCs is a better parameter than the degree of positivity, assuming proper gating of positive and negative cells [18, 63]. The background level of up to 6% (mean + 2 SD) positive RBCs set in our study is similar to that of others [60]. Because addition of antiglobulin to sub-agglutinating antibody-coated RBCs promote agglutination, finding the correct balance for antiglobulin binding without inducing agglutination is tricky and requires constant monitoring during FLOW DAT performance. As flow cytometry of erythrocytes is laborious, requires an expensive tool, and proves difficult to perform due to the tendency of erythrocytic agglutination, and FLOW DAT results do not appear to be superior to those with other DAT methods used in this study, it is unlikely that this technique will replace other classical DAT methods under most settings [66, 67].

We used two well-known polyvalent antisera against IgG, IgM, and C3 generated in either goat or rabbit, and achieved very similar results by the MICRO, CAPIL, and GEL LAB DAT methods in this study. However, for FLOW DAT, the polyclonal antiglobulins appeared to produce nonspecific irreproducible results during set-up, prompting us to use an IgG-specific antiglobulin reagent. A previous study reported that, in comparison to IgG alone, the use of IgG and IgM together dropped the specificity from 88 to 74%, while the sensitivity stayed at 88% [60]. Those findings compare well with the data of this study.

The most common antiglobulin reagent and method reported in the literature is the microtiter plate DAT assay with GAD [3, 11, 59]. The sensitivity of this reagent ranged from 61 to 95% in the literature, and the specificity ranged between 85 and 100% [3, 11, 59]. While the difference between antiglobulin reagents used for MICRO DAT are negligible, in two other DAT assays (CAPIL and GEL LAB), RAD seemed to be more sensitive and GAD seemed to be more specific. The overall κ values in this study should encourage the use of RAD for CAPIL and GAD for GEL LAB.

Our DAT+ results with the *anti-DEA 4* and *anti-Dal* antibodies and DAT- results with *anti-DEA 5* in blood samples of control dogs with a *DEA 4* + *, DEA 5-,* and *Dal* + blood type support the specificity of these DAT techniques and antisera to detect RBCs coated with antibodies. The inclusion of DAT+ and DAT- controls is important, and was also used in our prior DAT study [3], but it is rarely reported anywhere else. We realize that in vitro agglutination and binding reactions to alloantibodies may differ from that induced by autoantibodies in IMHA, but reagents consisting of canine autoantibodies are not available.

The main concern in IMHA are warm antibodies which are examined at body or room temperature. As the DAT results of this study were very similar if performed at 22°C or 37°C, it appears sufficient to set up the simpler room temperature incubation. Cold-antibody-mediated IMHA is extremely rare in humans and has not been documented in dogs. Thus, DAT at 4°C is rarely performed in clinical practice. However, cold agglutinins may cause agglutination in extremities and thereby may result in ear, fingertip, nail, and tail necrosis [8, 68].

The MICRO DAT and FLOW techniques allow for quantitation of DAT results, while all other DAT methods give only semiquantitative results. Serial dilution of the antiglobulin reagent is recommended to overcome a prozone effect caused by an excess of antiglobulin concentration. And if serial dilutions are not performed samples could be falsely labelled DAT-. Notably a prozone effect at lower dilutions (Fig. 2A) was seen in one fifth of the samples tested here. However, we observed a prozone effect only in those samples with high antiglobulin titers in the MICRO DAT, using either antiglobulin. While many publications on DAT refer to the prozone effect, there are few studies in dogs documenting the degree and frequency of the prozone effect in canine blood samples [62]. Interestingly, samples with an apparent prozone effect always produced DAT+ results with the other DAT methods which use fixed concentrations of antiglobulin. This finding is in concordance with our earlier report [3] as well as those in human medicine [62]. Therefore, these DAT methods with fixed antiglobulin quantities simplify the testing process and still permit a semi-quantitative assessment. Indeed, it was encouraging to find in this comparative study that the two in-clinic DAT techniques produced similar results to the more elaborated laboratory DAT methods.

To date, there is no clinical evidence that a higher MICRO DAT titer, number of DAT+ RBCs by FLOW or higher scores of the semiquantitative DAT methods are associated with a more severe clinical presentation, worse prognosis, and/or worse response of dogs with IMHA to treatment. In the present study, there was only a fair correlation between degree of DAT positivity among tests. Likewise, the degree of DAT positivity did not appear to be associated with the degree of anemia, erythroid regeneration, agglutination, or spherocytosis in this study. However, titers and semiquantitative scores may be helpful in monitoring disease and response to treatment.

### Follow-up test results of DAT+ cases

There is a paucity of data on dogs with IMHA during and after treatment [3, 24, 69], with most information relating to complications and survival after varied treatments of IMHA and rarely including any (immuno-) hematological test results [3, 24, 46, 69]. It is possible that veterinarians assume that DAT results will turn immediately negative after starting immunosuppressive treatment, even if dogs remain anemic and hemolysis is unresolved. Moreover, many clinicians assume that blood transfusions will cause an immediate positive DAT result [6], despite the fact that dogs have no clinically important naturally occurring alloantibodies [70]. Finally, a positive DAT is rarely if ever observed in previously transfused dogs, even in those with acute hemolytic transfusion reactions [71].

Based upon experience in human medicine and our prior experience, dogs with IMHA continue to be DAT+ until the antibody-mediated hemolysis resolves. As in our prior studies [3, 24], nine of 12 DAT+ dogs, that we were able to follow by testing, remained DAT+ for weeks. Among the 12 DAT+ dogs monitored, 10 were no longer anemic when tested and in most cases their macroscopic agglutination and spherocytosis had resolved suggesting clinical improvement (was also the assessment by attending clinicians) while in part still being DAT+ . Moreover, the 42 attending clinicians who responded reported that 70% of the DAT+ dogs with IMHA recovered or were clinically stable, while 30% of them died or were euthanized. These findings concur with prior outcome surveys [19, 72]. Thus, while the results of the follow-up data are sparse in this as well as in prior studies [3, 24, 25, 44, 69], they support the value of monitoring dogs with IMHA during and after treatment. Thus, the authors encourage clinicians to not only monitor the degree of anemia, but to also follow the erythroid regenerative response, autoagglutination, spherocytosis, and DAT when treating dogs with IMHA.

### Study limitations

Samples were shipped to a large commercial laboratory and stored refrigerated for 2–5 days before analysis. Although this delay may have introduced artifacts, it reflects the clinical reality. Interestingly, agglutination, spherocytosis, and DAT results could still be well assessed, despite the delays. While fresh samples are certainly preferred for any morphological, immunological, and functional RBC studies, our study suggests that delays in testing of samples kept refrigerated do not hamper or preclude receiving meaningful positive immunohematological results. While some investigators had claimed that the DAT could only be done on fresh samples [16], our previous study [3] also indicated that the DAT results remain meaningful for blood that had been stored refrigerated for days. We have clinical follow-up information on only 42 of 67 DAT+ dogs, and they were clinically assessed to have IMHA by the attending clinician. In addition, we have supportive routine and immunohematological results for those as well as all other DAT+ dogs. As in other studies, we do not definitively know if any of the 126 dogs had or did not have IMHA. Finally, we did not investigate whether it was a primary (non-associative) or secondary (associative) IMHA.

The immunodiagnostic techniques were specifically established in one major veterinary diagnostic laboratory setting and performed each time by the same individual under essentially identical conditions. While this meant that the operator was not blinded to the other test results, every effort was made to objectively analyze each test result; and many results were and could be captured by photography for later review.

## Conclusions

In this large cohort of 126 dogs suspected of having IMHA based on clinical data, CBC, and chemistry panel and 28 healthy control dogs, we obtained similar and consistent results using various DAT methods. There was also a good correlation between DAT and marked spherocytosis, but only fair agreement between DAT and agglutination, including SAT. The DAT methods, were both analytically more sensitive and more specific, detecting additional dogs with antibody-coated erythrocytes and excluding nonspecific agglutination. Furthermore, macroscopic autoagglutination only rarely interfered with interpretation of DAT results. Finally, DAT+ dogs revealed DAT+ results that persisted for weeks. This suggests that DAT might be useful in monitoring dogs receiving treatment for IMHA.This prospective study is the most comprehensive clinical comparative investigation of DAT methods to date, and showed excellent correlations between DAT methods. We hope that our findings will restore veterinary clinicians’ confidence in DAT methods and results.

## Supplementary Information

**Additional file 1: Supplementary Table S1.** Test conditions for six direct antiglobulin test (DAT) methods. **Supplementary Table S2.** Demographic data of 126 dogs suspected to have immune-mediated hemolytic anemia (IMHA) and 28 healthy control dogs and with DAT+ results by microtiter. Plate direct antiglobulin test (DAT) with using goat anti-dog IgG, IgM, and C3 at 22°C. Breed (A), age (B), and gender (C) distribution. **Supplementary Tables S3.** Demographic data of 126 dogs suspected to have immune-mediated hemolytic anemia (IMHA) with positive and negative direct antiglobulin test (DAT) results. Breed (A), age (B), and gender (C) distribution. **Supplementary Table S4.** *In vitro*-induced positive direct antiglobulin test (DAT) results in samples from eight healthy dogs with *DEA 4+*, *DEA 5-* and *Dal+* blood type by adding *anti-DEA 4, anti-DEA 5*, and *anti-Dal* antisera. **Supplementary Table S5.** Anemia, reticulocytosis, hyperbilirubinemia, and hemolyzed plasma compared to DAT results in dogs suspected to have IMHA. **Supplementary Table S6.** Degree of anemia (total hemoglobin [Hb], packed cell volume [PCV], and hematocrit [Hct]) in relation to direct antiglobulin test (DAT) results in dogs suspected to have immune-mediated hemolytic anemia. **Supplementary Table S7.** Correlation between polychromasia and reticulocytosis of samples from 80 dogs suspected to have immune-mediated hemolytic anemia and MICRO GAD direct antiglobulin test. Results from DAT+ dogs in brackets. **Supplementary Table S8.** Microtiter plate direct antiglobulin test (DAT) results with two antiglobulins (goat anti-dog; GAD and rabbit anti-dog; RAD) and at three different temperatures (22, 37 and 4°C) for 126 dogs suspected to have immune-mediated hemolytic anemia. **Supplementary Table S9.** Comparison of six different direct antiglobulin tests (DAT) with one to two different antiglobulin reagent. **Supplementary Table S10.** Minitube gel kit direct antiglobulin test (GEL KIT DAT) in dogs suspected to have immune-mediated hemolytic anemia with and without prior 3x washing of red blood cells. **Supplementary Table S11.** Comparison of six direct antiglobulin test (DAT) results in 126 dogs suspected to have immune-mediated hemolytic anemia (IMHA). The Cohen’s kappa (κ) values and a two-sided confidence intervals (95%) is reported for each pair of methods used. **Supplementary Table S12.** Comparison of six direct antiglobulin test (DAT) results in 126 dogs suspected to have immune-mediated hemolytic anemia (IMHA) and 28 healthy dogs. The Cohen’s kappa (κ) values and two-sided confidence intervals (95%) are reported for each pair of methods used. **Supplement Table S13.** Cohen’s kappa (κ) value of the spherocytosis, and macroscopic in tube agglutination and macroscopic saline agglutination test (SAT) results at two dilutions compared to the microtiter plate DAT method with goat-anti dog IgG, IgM, and C3 at 22°C. **Supplementary Figure S1.** Flow chart oflaboratory tests and six different direct antiglobulin test (DAT) methods performed with blood samples from dogs suspected to have immune-mediated hemolytic anemia. **Supplementary Figure S2.** Severity of anemia, reticulocytosis, hyperbilirubinemia, and free plasma hemoglobin concentration compared to microtiter plate direct antiglobulin test (MICRO DAT) results in dogs suspected to have immune-mediated hemolytic anemia. **Supplementary Figure S3.** Severity of anemia, reticulocytosis, bilirubinemia, and free plasma hemoglobin concentration results grouped based on ≥4 DAT+ results in dogs suspected to have immune-mediated hemolytic anemia

## Data Availability

The datasets used and/or analyzed during the current study are available from the corresponding author on reasonable request.

## Abbreviations

ACVIM: American College of Veterinary Internal Medicine
BSA: Bovine serum albumin
CAPIL: Microcapillary tube
CAPIL GAD: Microcapillary tube with goat anti-dog IgG, IgM, and C3 antiglobulin
CAPIL RAD: With rabbit anti-dog IgG, IgM, and C3 antiglobulin
C3: Complement component 3
CBC: Complete blood count
CI: Confidence interval
DAT+ /DAT-: Direct antiglobulin test (with positive/negative result)
DEA: Dog erythrocyte antigen
EDTA: Ethylenediaminetetraacetic acid
FITC: Fluorescein isothiocyanate
FLOW: Flow cytometry
FITC-GAD: FITC-marked goat anti-dog IgG (heavy and light chains)
GAD: Goat anti-dog IgG, IgM, and C3
GEL KIT: In-clinic gel minitube kit
GEL LAB: Neutral gel column card with added antiglobulin
GEL LAB GAD: Neutral gel column card with goat anti-dog IgG, IgM, and C3 antiglobulin
GEL LAB RAD: Neutral gel column card with rabbit anti-dog IgG, IgM, and C3 antiglobulin
Hct: Calculated hematocrit by hematology analyzer or 3 × Hb concentration in g/dL
Hb: Hemoglobin
hpf: High-power field
h: Hour(s)
IAT: Indirect antiglobulin test
IgG and IgM: Immunoglobulin G and M
IMHA: Immune-mediated hemolytic anemia
MFI: Mean fluorescence intensity
MICRO: Microtiter plate
MICRO GAD: Microtiter plate with goat anti-dog IgG, IgM, and C3 antiglobulin
MICRO RAD: Microtiter plate with rabbit anti-dog IgG, IgM, and C3 antiglobulin
min: Minute(s)
PBS: Phosphate-buffered saline
PCV: Packed cell volume
pRBCs: Packed red blood cells
RAD: Rabbit anti-dog IgG, IgM, and C3
RBC(s): Red blood cell(s)
SAT: Saline agglutination test
SD/SE: Standard deviation/error of the mean
STRIP KIT: In-clinic immunochromatographic strip kit
