# Supplementary material for: Comparative study of immunohematological tests with canine blood samples submitted for a direct antiglobulin (Coombs’) test
Source: Canine Med Genet. 2021 Oct 13;8:10. doi: 10.1186/s40575-021-00107-0 (PMC8515749; doi:10.1186/s40575-021-00107-0)
Supplement: Supplementary file 1 — Additional file 1: Supplementary Table S1. Test conditions for six direct antiglobulin test (DAT) methods. Supplementary Table S2. Demographic data of 126 dogs suspected to have immune-mediated hemolytic anemia (IMHA) and 28 healthy control dogs and with DAT+ results by microtiter. Plate direct antiglobulin test (DAT) with using goat anti-dog IgG, IgM, and C3 at 22°C. Breed (A), age (B), and gender (C) distribution. Supplementary Tables S3. Demographic data of 126 dogs suspected to have immune-mediated hemolytic anemia (IMHA) with positive and negative direct antiglobulin test (DAT) results. Breed (A), age (B), and gender (C) distribution. Supplementary Table S4. In vitro-induced positive direct antiglobulin test (DAT) results in samples from eight healthy dogs with DEA 4+, DEA 5- and Dal+ blood type by adding anti-DEA 4, anti-DEA 5, and anti-Dal antisera. Supplementary Table S5. Anemia, reticulocytosis, hyperbilirubinemia, and hemolyzed plasma compared to DAT results in dogs suspected to have IMHA. Supplementary Table S6. Degree of anemia (total hemoglobin [Hb], packed cell volume [PCV], and hematocrit [Hct]) in relation to direct antiglobulin test (DAT) results in dogs suspected to have immune-mediated hemolytic anemia. Supplementary Table S7. Correlation between polychromasia and reticulocytosis of samples from 80 dogs suspected to have immune-mediated hemolytic anemia and MICRO GAD direct antiglobulin test. Results from DAT+ dogs in brackets. Supplementary Table S8. Microtiter plate direct antiglobulin test (DAT) results with two antiglobulins (goat anti-dog; GAD and rabbit anti-dog; RAD) and at three different temperatures (22, 37 and 4°C) for 126 dogs suspected to have immune-mediated hemolytic anemia. Supplementary Table S9. Comparison of six different direct antiglobulin tests (DAT) with one to two different antiglobulin reagent. Supplementary Table S10. Minitube gel kit direct antiglobulin test (GEL KIT DAT) in dogs suspected to have immune-mediated hemolyt [file 40575_2021_107_MOESM1_ESM.docx]

**SUPPLEMENT**

| **DAT method** | **Washed pRBCs** | **Added Fluid Volume** | **RBC Suspension** | **Antiglobulin** | **RBC Suspension : Antiglobulin Volume** | **Incubation Conditions** | **Centrifugation** |
| --- | --- | --- | --- | --- | --- | --- | --- |
| In-clinic strip kit^1^ | 5 µL | 200 µL saline | 2.5 % | RAD | 205 µL: 0 µL | 5-15 min 22°C | ND |
| In-clinic gel kit^1^ | 5 µL* | 500 µL saline | 1 % | RAD | 30 µL : 0 µL | ND | 1x 10 min 200 g |
| Neutral gel column card | 5 µL* | 500 µL saline | 1 % | RAD or GAD | 12.5 µL : 6.25 µL | 15 min 37°C | 1x 10 min 85 g |
| Microtiter plate | 14 µL | 345 µL saline | 4 % | RAD or GAD | 15 µL : 15 µL | 30 min 22°C | ND |
| Microcapillary tube | 12 µL | 40 µL saline | 30 % | RAD or GAD | 3 µL : 3 µL | 10 min 22°C | ND |
| Flow cytometry | 5 µL | 500 µL BSA-PBS | 1 % | GAD-IgG# | 100 µL: 100 µL | 30 min 4°C | 3 x 15 min 200 g |

**Supplementary Table S1** Test conditions for six direct antiglobulin test (DAT) methods

*with and without washing; ^1^Product from Alvedia, Lyon, France; #FITC-marked goat anti-dog IgG (H+L); BSA-PBS: bovine serum albumin phosphate-buffered saline;
GAD: goat anti-dog IgG, IgM, and C3 antiglobulin (VMRD, Pullman, WA, USA); RAD: rabbit anti-dog IgG, IgM, and C3 antiglobulin (MP Biomedicals, Solon, OH, USA); ND: Not done

**Supplementary Tables S2** Demographic data of 126 dogs suspected to have immune-mediated hemolytic anemia (IMHA) and 28 healthy control dogs with DAT+ results by microtiter
 plate direct antiglobulin test (DAT) using goat anti-dog IgG, IgM, and C3 at 22°C. Breed (A), age (B), and gender (C) distribution

**C**

**A**

**B**

| **Age** | | | |
| --- | --- | --- | --- |
| **Age  (years)** | **Control  Dogs** | **Suspect IMHA Dogs** | **DAT+ Dogs** |
| <1 | 1 | 4 | 2 |
| 1 | 2 | 5 | 4 |
| 2 | 2 | 5 | 4 |
| 3 | 2 | 4 | 2 |
| 4 | 1 | 8 | 6 |
| 5 | 1 | 11 | 5 |
| 6 | 3 | 11 | 5 |
| 7 | 1 | 11 | 5 |
| 8 | 0 | 9 | 4 |
| 9 | 4 | 17 | 12 |
| 10 | 1 | 8 | 5 |
| 11 | 2 | 13 | 4 |
| 12 | 2 | 6 | 5 |
| 13 | 3 | 3 | 1 |
| 14 | 2 | 3 | 1 |
| 15 | 0 | 2 | 0 |
| Not reported | 1 | 6 | 2 |
| Range | <1-14 | <1-15 | <1-15 |
| Median | 9 | 8 | 7 |
| Total | 28 | 126 | 67 |

| **Breed** | | | |
| --- | --- | --- | --- |
| **Breed** | **Control  Dogs** | **Suspect IMHA  Dogs** | **DAT+ Dogs** |
| Mix | 20 | 44 | 26 |
| Jack Russel Terrier | 0 | 6 | 2 |
| Cocker Spaniel | 0 | 4 | 3 |
| Labrador Retriever | 0 | 4 | 3 |
| Maltese | 0 | 4 | 4 |
| Rhodesian Ridgeback | 0 | 4 | 0 |
| German Shepherd | 0 | 3 | 1 |
| Havanese | 0 | 3 | 3 |
| Irish Setter | 0 | 3 | 2 |
| Vizsla | 0 | 3 | 1 |
| Australian Shepherd | 0 | 2 | 0 |
| Cavalier King Charles Spaniel | 0 | 2 | 0 |
| Dachshund | 0 | 2 | 1 |
| Fox Terrier | 0 | 2 | 2 |
| Husky | 0 | 2 | 1 |
| Poodle | 0 | 2 | 2 |
| Rottweiler | 0 | 2 | 0 |
| Saint-Bernard | 0 | 2 | 0 |
| Weimaraner | 0 | 2 | 1 |
| Border Collie | 3 | 1 | 1 |
| Others (one dog per breed) | 5 | 25 | 12 |
| Not reported | 0 | 4 | 2 |
| Total | 28 | 126 | 67 |

| **Sex** | | | |
| --- | --- | --- | --- |
| **Sex** | **Control  Dogs (%)** | **Suspect IMHA  Dogs (%)** | **DAT+ Dogs (%)** |
| Female | 17 (61) | 69 (55) | 43 (64) |
| Male | 11 (39) | 49 (39) | 18 (27) |
| Not reported | 0 (0) | 8 (6) | 6 (9) |
| Total | 28 | 126 | 67 |

Groups were compared by Mann-Whitney U test and chi-squared test.
All samples from controls dogs were DAT- by all methods and showed neither agglutination nor spherocytosis.
The sex was significantly different between the MICRO GAD 22°C DAT+ and DAT- group: χ^2^ (1) = 7.5, p = 0.006.
There was no statistically significant different in age between MICRO GAD 22°C DAT+ and DAT-: U = 1555.0, Z = -1.2, p = .220.
There is no statistical difference in age between the control group and the test group: U = 1550.0, Z = -.3, p = .725.
There is no statistical difference in sex between the control group and the test group: χ^2^ (1) = 0.4, p = .829.

**Supplementary Table S3** Demographic data of 126 dogs suspected to have immune-mediated hemolytic anemia (IMHA) with positive and negative direct antiglobulin test (DAT) results. Breed (A), age (B), and gender (C) distribution

| **Breed** | | |
| --- | --- | --- |
| **Breed** | **≥4 DAT+ Dogs** | |
|  | **Yes** | **No** |
| Mix | 26 | 17 |
| Havanese | 3 | 0 |
| Jack Russel Terrier | 3 | 3 |
| Labrador Retriever | 3 | 1 |
| Maltese | 3 | 1 |
| Cocker Spaniel | 2 | 1 |
| Fox Terrier | 2 | 0 |
| German Shepherd | 2 | 1 |
| Irish Setter | 2 | 1 |
| Poodle | 2 | 0 |
| Australian Shepherd | 0 | 2 |
| Cavalier King Charles Spaniel | 0 | 2 |
| Magyar Viszla | 0 | 2 |
| Rhodesian Ridgeback | 0 | 4 |
| Rottweiler | 0 | 2 |
| Saint-Bernard | 0 | 2 |
| Others (one dog per breed) | 18 | 25 |
| Not reported | 1 | 3 |
| Total | 67 | 59 |

| **Age** | | |
| --- | --- | --- |
| **Age** | **≥4 DAT+ Dogs** | |
| **(years)** | **Yes** | **No** |
| <1 | 2 | 2 |
| 1 | 1 | 1 |
| 2 | 3 | 2 |
| 3 | 2 | 2 |
| 4 | 7 | 1 |
| 5 | 5 | 6 |
| 6 | 6 | 7 |
| 7 | 5 | 6 |
| 8 | 4 | 5 |
| 9 | 12 | 5 |
| 10 | 4 | 4 |
| 11 | 7 | 6 |
| 12 | 4 | 2 |
| 13 | 1 | 2 |
| 14 | 1 | 2 |
| 15 | 0 | 2 |
| Not reported | 2 | 4 |
| Range | <1-14 | <1-15 |
| Median | 8 | 8 |
| Total | 67 | 59 |

**C**

**A**

**B**

| **Sex** | | |
| --- | --- | --- |
| **Sex** | **≥4 DAT+ Dogs (%)** | |
|  | **Yes** | **No** |
| Female | 40 (60) | 29 (49) |
| Male | 22 (33) | 27 (46) |
| Not reported | 5 (7) | 3 (5) |
| Total | 67 | 59 |

Groups were compared by Mann-Whitney U test and chi-squared test.
The sex was not significantly different in the ≥4 DAT+ versus DAT- group: χ^2^ (1) = 1.9, p = 0.16.
There was no statistically significant difference in age between ≥4 DAT+ and DAT-: U = 1623.5, Z = -.8, p = .388.

**Supplementary Table S4** *In vitro*-induced positive direct antiglobulin test (DAT) results in samples from eight healthy dogs with
*DEA 4+, DEA 5-* and *Dal+* blood type by adding *anti-DEA 4, anti-DEA 5*, and *anti-Dal* antisera

| **Dog #** | **GEL LAB DAT** | | |  | **CAPIL DAT** | | |  | **MICRO DAT** | | |
| --- | --- | --- | --- | --- | --- | --- | --- | --- | --- | --- | --- |
|  | *Anti-DEA 4* | *Anti-DEA 5* | *Anti-Dal* |  | *Anti- DEA 4* | *Anti- DEA 5* | *Anti-Dal* |  | *Anti- DEA 4* | *Anti- DEA 5* | *Anti- Dal* |
| 1 | 4+ | - | 4+ |  | + | - | + |  | 1:8 | - | 1:256 |
| 2 | 4+ | - | 4+ |  | + | - | + |  | 1:32 | - | 1:256 |
| 3 | 4+ | - | 4+ |  | + | - | + |  | 1:128 | - | 1:1024 |
| 4 | 4+ | - | 4+ |  | + | - | + |  | 1:16 | - | 1:256 |
| 5 | 4+ | - | 4+ |  | + | - | + |  | 1:8 | - | 1:512 |
| 6 | 4+ | - | 4+ |  | + | - | + |  | 1:16 | - | 1:1024 |
| 7 | 4+ | - | 4+ |  | + | - | + |  | 1:16 | - | 1:256 |
| 8 | 4+ | - | 4+ |  | + | - | + |  | 1:32 | - | 1:256 |

GEL LAB: neutral gel column card DAT; CAPIL: microcapillary DAT; MICRO: microtiter plate DAT tested at 22°C, 37°C and 4°C

There was no marked titer difference at any temperature, and only the lowest titer of the three temperatures is reported in table.

**Supplementary Table S5** Anemia, reticulocytosis, hyperbilirubinemia, and hemolyzed plasma compared to DAT results in dogs suspected to have IMHA

| **Test Group** | **DAT  Result** |  | **Anemia**  (n = 123**)** | |  | **Reticulocytosis** (n = 84) | |  | **Hyperbilirubinemia**  (n = 60) | |  | **Hemolyzed**  (n = 93) | |
| --- | --- | --- | --- | --- | --- | --- | --- | --- | --- | --- | --- | --- | --- |
|  |  |  | Yes | No |  | Yes | No |  | Yes | No |  | Yes | No |
|  |  |  |  |  |  |  |  |  |  |  |  |  |  |
| **MICRO DAT+** | Yes |  | 65 | 2 |  | 32 | 15 |  | 27 | 4 |  | 41 | 5 |
|  | No |  | 41 | 15 |  | 17 | 20 |  | 7 | 22 |  | 34 | 13 |
|  | *p value* |  | ***< .001*** | |  | ***.048*** | |  | ***< .001*** | |  | *.065* | |
|  |  |  |  | |  |  | |  |  | |  |  | |
|  |  |  |  |  |  |  |  |  |  |  |  |  |  |
| **≥4 DAT+** | Yes |  | 65 | 2 |  | 32 | 17 |  | 28 | 6 |  | 41 | 5 |
|  | No |  | 41 | 15 |  | 17 | 18 |  | 6 | 20 |  | 34 | 13 |
|  | *p value* |  | ***< .001*** | |  | *.178* | |  | ***< .001*** | |  | *.065* | |
|  |  |  |  | |  |  | |  |  | |  |  | |

n: number of dogs tested and included in analysis. Data was not available for all samples.
Groups were compared by chi-squared test.
MICRO DAT: microtiter plate direct antiglobulin test with goat anti-dog IgG, IgM, and C3 reagent incubated at 22°C
≥4 DAT+: at least four different direct antiglobulin test results were positive among the five to six performed DATs
Anemia defined as PCV <36% (available in 82 cases, calculated from the hemoglobin in EDTA-blood in 41 cases); reticulocytosis; >110,000/nL; hyperbilirubinemia >3.4 µmol/L; hemolyzed plasma >0.1 g/dL hemoglobin
DAT+/DAT-: direct antiglobulin test positive/negative results

**Supplementary Table S6** Degree of anemia (total hemoglobin [Hb], packed cell volume [PCV], and hematocrit [Hct]) in relation to direct antiglobulin test (DAT) results
 in dogs suspected to have immune-mediated hemolytic anemia

|  |  |  | **Total Hemoglobin, Hb (n=115)** | | | |  | **Packed Cell Volume, PCV (n=82)** | | | |  | **Hematocrit, Hct (n=84)** | | | |
| --- | --- | --- | --- | --- | --- | --- | --- | --- | --- | --- | --- | --- | --- | --- | --- | --- |
| ***Anemia*** |  |  | *none* | *mild* | *mod* | *sev* |  | *none* | *mild* | *mod* | *sev* |  | *none* | *mild* | *mod* | *sev* |
|  |  |  |  |  |  |  |  |  |  |  |  |  |  |  |  |  |
| MICRO DAT+ | No |  | 18 | 7 | 24 | 8 |  | 12 | 9 | 15 | 5 |  | 9 | 14 | 7 | 6 |
|  | Yes |  | 2 | 2 | 27 | 27 |  | 1 | 0 | 14 | 26 |  | 1 | 4 | 28 | 15 |
| **All cases** |  |  | **20** | **9** | **51** | **35** |  | **13** | **9** | **29** | **31** |  | **10** | **18** | **35** | **21** |
|  |  |  |  |  |  |  |  |  |  |  |  |  |  |  |  |  |
|  |  |  |  |  |  |  |  |  |  |  |  |  |  |  |  |  |
| ≥4 DAT+ | No |  | 17 | 7 | 23 | 10 |  | 12 | 8 | 16 | 7 |  | 9 | 13 | 7 | 5 |
|  | Yes |  | 3 | 2 | 28 | 25 |  | 1 | 1 | 13 | 24 |  | 1 | 5 | 28 | 16 |
| **All cases** |  |  | **20** | **9** | **51** | **35** |  | **13** | **9** | **29** | **31** |  | **10** | **18** | **35** | **21** |
|  |  |  |  |  |  |  |  |  |  |  |  |  |  |  |  |  |

MICRO DAT: microtiter plate direct antiglobulin test with goat anti-dog IgG, IgM, and C3.
<4 DAT+: positive with at least four DAT methods from the five tested (excluding flow cytometry).
None: no anemia (>36%); mild: mild anemia (30-36%); mod: moderate anemia (18-29%); sev: severe anemia (<18%)
Groups were compared by Kruskal-Wallis test:
The severity of the anemia (total Hb) in MICRO DAT- (mean rank = 43.3) and MICRO DAT+ (mean rank = 72.4) differ H(1) = 25.0, p < .001.
The severity of the anemia (PCV) in MICRO DAT- (mean rank = 27.7) and MICRO DAT+ (mean rank = 55.2) differ, H(1) = 30.6, p < .001.
The severity of the anemia (Hct) in MICRO DAT- (mean rank = 30.2) and MICRO DAT+ (mean rank = 51.7) differ, H(1) = 17.6, p < .001.
The severity of the anemia (total Hb) in DAT- (mean rank = 45.5) and <4 DAT+ (mean rank = 70.2) differ, H(1) = 17.8, p < .001.
The severity of the anemia (PCV) in <4 DAT- (mean rank = 29.9) and <4 DAT+ (mean rank = 54.2) differ, H(1) = 23.6, p < .001.
The severity of the anemia (Hct) in <4 DAT- (mean rank = 29.2) and <4 DAT+ (mean rank = 51.5) differ, H(1) = 18.6, p < .001.

**Supplementary Table S7** Correlation between polychromasia and reticulocytosis of samples from 80 dogs suspected to have immune-mediated hemolytic anemia and MICRO GAD direct antiglobulin test. Results from DAT+ dogs in brackets

| N=80 (43) |  | **Polychromasia** | |  |
| --- | --- | --- | --- | --- |
|  |  | Yes | No | |
| **Reticulocytosis** | Yes | 41 (28) | 6 (2) | |
|  | No | 5 (1) | 28 (12) | |

There is an association between polychromasia (1+ to 4+) and reticulocytosis (>110,000/nL).

Groups were compared by Fischer’s test: p < .001

**Supplementary Table S8** Microtiter plate direct antiglobulin test (DAT) results with two antiglobulins (goat anti-dog; GAD and rabbit anti-dog; RAD) and at three different temperatures (22°C, 37°C, and 4°C) for 126 dogs suspected to have immune-mediated hemolytic anemia

|  |  | **MICRO GAD** | | | | | | | | |  | **MICRO RAD** | | | | | | | | | | | | | | | | | | |  |
| --- | --- | --- | --- | --- | --- | --- | --- | --- | --- | --- | --- | --- | --- | --- | --- | --- | --- | --- | --- | --- | --- | --- | --- | --- | --- | --- | --- | --- | --- | --- | --- |
|  |  | **37°C** | | | |  | **4°C** | | | |  | | **37°C** | | | | |  | | **22°C** | | | | |  | | **4°C** | | | | |
| **MICRO GAD DAT 22°C** | From | 0 | 8 | 128 | 1024 |  | 0 | 8 | 128 | 1024 |  | 0 | | 8 | 128 | 1024 |  | | 0 | | 8 | 128 | 1024 |  | | 0 | | 8 | 128 | 1024 |  |
|  | to | 4 | 64 | 512 | 2048 |  | 4 | 64 | 512 | 2048 |  | 4 | | 64 | 512 | 2048 |  | | 4 | | 64 | 512 | 2048 |  | | 4 | | 64 | 512 | 2048 |  |
|  | ≤ 4 | 57 | 1 | 1 | 0 |  | 57 | 1 | 1 | 0 |  | 54 | | 2 | 3 | 0 |  | | 57 | | 0 | 2 | 0 |  | | 55 | | 2 | 2 | 0 |  |
|  | 8-64 | 0 | 3 | 0 | 0 |  | 0 | 3 | 0 | 0 |  | 0 | | 3 | 0 | 0 |  | | 0 | | 3 | 0 | 0 |  | | 0 | | 3 | 0 | 0 |  |
|  | 128-512 | 0 | 1 | 6 | 3 |  | 0 | 3 | 3 | 4 |  | 1 | | 3 | 5 | 1 |  | | 2 | | 2 | 6 | 0 |  | | 2 | | 2 | 3 | 3 |  |
|  | 1024-2048 | 1 | 0 | 4 | 49 |  | 2 | 1 | 6 | 45 |  | 2 | | 5 | 9 | 38 |  | | 1 | | 2 | 4 | 47 |  | | 3 | | 6 | 9 | 36 |  |

Agglutination titers < 1:4 were considered DAT-
Groups were compared by Wilcoxon Signed-Ranks test:
MICRO GAD 22°C (mean rank = 6.0) and MICRO GAD 37°C (mean rank = 6.0) do not differ, Z = -.2, p = .842.
MICRO GAD 22°C (mean rank = 9.8) and MICRO GAD 4°C (mean rank = 8.8) do not differ, Z = -1.4, p = .156.
MICRO GAD 22°C (mean rank = 13.3) and MICRO RAD 37°C (mean rank = 14.0) do not differ, Z = -2.3, p = .0.16.
MICRO GAD 22°C (mean rank = 6.5) and MICRO RAD 22°C (mean rank = 9.5) do not differ, Z = -1.8, p = .053.
MICRO GAD 22°C (mean rank = 15.8) and MICRO RAD 4°C (mean rank = 12.2) differ, Z = -2.9, p = .003. The effect size r is small, r = 0.26.

| **MICRO DAT titer** | **GEL KIT  RAD** | | |  | **GEL LAB  GAD/RAD** | | | | |  | **STRIP KIT  RAD** | | | | |  | **CAPIL GAD/RAD** | |  | **FLOW  GAD*** | | |
| --- | --- | --- | --- | --- | --- | --- | --- | --- | --- | --- | --- | --- | --- | --- | --- | --- | --- | --- | --- | --- | --- | --- |
|  | - | 3+ | 4+ |  | - | 1+ | 2+ | 3+ | 4+ |  | - | 1+ | 2+ | 3+ | 4+ |  | - | + |  | - | <50% | >50% |
| <1:4 | 49 | 1 | 9 |  | 54/48 | 3/7 | 0/0 | 0/1 | 2/3 |  | 46 | 8 | 3 | 0 | 2 |  | 55/53 | 4/6 |  | 16 | 10 | 0 |
| 1:8 - 1:64 | 0 | 0 | 3 |  | 2 / 2 | 0/0 | 0/1 | 0/0 | 1/0 |  | 0 | 0 | 1 | 0 | 2 |  | 1/1 | 2/2 |  | 0 | 1 | 0 |
| 1:128 - 1:512 | 0 | 0 | 10 |  | 4 / 3 | 0/1 | 0/0 | 1/1 | 5/5 |  | 0 | 4 | 1 | 1 | 4 |  | 3/2 | 7/8 |  | 0 | 4 | 2 |
| 1:1024 -1:2048 | 3 | 1 | 50 |  | 14/12 | 7/4 | 4/6 | 8/7 | 21/25 |  | 1 | 9 | 16 | 13 | 15 |  | 12/2 | 42/52 |  | 2 | 19 | 15 |
|  |  |  |  |  |  |  |  |  |  |  |  |  |  |  |  |  |  |  |  |  |  |  |
| All cases | 51 | 2 | 72 |  | 73/64 | 10/12 | 4/7 | 9/9 | 29/33 |  | 46 | 21 | 21 | 14 | 23 |  | 70/57 | 55/68 |  | 18 | 34 | 17 |

**Supplementary Table S9** Comparison of six different direct antiglobulin tests (DAT) with one to two different antiglobulin reagent

DAT methods: STRIP: in-clinic immunochromatographic strip kit; FLOW: flow cytometry (n = 69); CAPIL: microcapillary tube; MICRO: microtiter plate; GEL KIT: in-clinic gel minitube kit; GEL LAB: neutral gel column card with added antiglobulin
Reagents: GAD: goat anti-dog IgG, IgM, and C3; RAD: rabbit anti-dog IgG, IgM, and C3, GAD*: FITC-marked goat anti-dog IgG (H+L)
MICRO: ≤1:4 was considered negative; for all other (-) is considered DAT- and for 1+ to 4+ DAT+.
Groups were compared by Wilcoxon Signed-Ranks test.
MICRO GAD 22°C (mean rank = 17.6) and GEL KIT RAD (mean rank = 13.3) do differ, Z = -2.9, p = .003. The effect size r is small, r = 0.26.
MICRO GAD 22°C (mean rank = 28.2) and GEL LAB GAD (mean rank = 15.7) do differ, Z = -4.5, p < .001. The effect size r is moderate, r = 0.40.
MICRO GAD 22°C (mean rank = 29.7) and GEL LAB RAD (mean rank = 17.7) do differ, Z = -3.5, p < .001. The effect size r is moderate, r = 0.31.
MICRO GAD 22°C (mean rank = 34.8) and STRIP KIT RAD (mean rank = 25.3) do differ, Z = -3.6, p< .001. The effect size r is moderate, r = 0.32.
MICRO GAD 22°C (mean rank = 10.5) and CAPIL GAD (mean rank = 10.5) do not differ, Z = -2.6, p = .12.
MICRO GAD 22°C (mean rank = 6.0) and CAPIL RAD (mean rank = 6.0) do not differ, Z = -.3, p = 1.
MICRO GAD 22°C (mean rank = 18.4) and FLOW GAD (mean rank = 17.0) do differ, Z = -2.6, p = .009. The effect size r is small, r = 0.23.

**Supplementary Table S10** Minitube gel kit direct antiglobulin test (GEL KIT DAT) in dogs suspected to have immune-mediated hemolytic anemia with and without prior 3x washing of red blood cells

| N=126 |  | **GEL KIT DAT**  **With Washing** | |  |
| --- | --- | --- | --- | --- |
|  |  | DAT- | DAT+ | |
| **GEL KIT DAT Without Washing** | DAT- | 52 | 0 | |
|  | DAT+ | 4* | 70 | |

* Two of four samples showed a double population, and the other two samples were weakly positive without washing and DAT- with washing
Groups were compared by Cohen’s kappa (κ) test, and there is a very good agreement between the two methods, κ = .93 (95% CI, .87 to .99), p <.001.
Interpretation of the Κ values: very good agreement (≥ .81); good agreement (≥ .61); moderate agreement (≥ .41); fair agreement (≥ .21), as per Landis and Koch [1] and adapted by Brennan and Silman [2]

| **DAT Method** | Antiglobulin  Temperature  Washing | **FLOW** | **CAPIL** | | **MICRO** | | | | | | **GEL KIT** | | **GEL LAB** | | | |
| --- | --- | --- | --- | --- | --- | --- | --- | --- | --- | --- | --- | --- | --- | --- | --- | --- |
|  |  | GAD*  22°C | GAD  22°C | RAD  22°C | GAD 22°C | GAD 37°C | GAD  4°C | RAD  22°C | RAD  37°C | RAD  4°C | RAD  22°C | RAD  22°C washed | GAD  22°C | GAD  22°C washed | RAD  22°C | RAD  22°C  washed |
| **STRIP KIT** | RAD 22°C | .66  .46-.86 | .56  .43-.70 | .72  .60-.84 | .77  .66-.88 | .78  .68-.89 | .74 .62-.85 | .75  .64-.87 | .80  .70-.90 | .75  .64-.87 | .75  .63-.86 | .72  .60-.84 | .70  .57-.82 | .52  .39-.66 | .47  .32-.62 | .46  .31-.61 |
| **FLOW** | GAD 22°C |  | .46  .26-.65 | .60  .41-.80 | .60  .41-.80 | .60  .41-.80 | .55  .35-.75 | .60  .41-.80 | .66  .47-.84 | .57  .38-.77 | .66  .47-.84 | .60  .41-.80 | .66  .47-.84 | .39  .20-.58 | .43  .18-.67 | .37  .18-.57 |
| **CAPIL** | GAD 22°C |  |  | .76  .65-.87 | .68  .56-.80 | .67  0.54-0.79 | .71  .59-.83 | 0.70  .57-.82 | .65  .52-.78 | .70  .57-.82 | .70  .58-.82 | 0.70  .58-.82 | .62  .49-.75 | .72  .60-.84 | .32  .20-.43 | .64  .51-.78 |
|  | RAD 22°C |  |  |  | .82  .72-.92 | .87  .78-.95 | .85  .76-.94 | .84  .74-.93 | .82  .72-.92 | .80  .70-.91 | .80  .70-.91 | .80  .70-.91 | .69  .56-.82 | .68  .56-.80 | .45  .31-.58 | .60  .46-.74 |
| **MICRO** | GAD 22°C |  |  |  |  | .95  .89-1.0 | .93  .97-.99 | .92  .85-.98 | .87  .78-.95 | .85  .76-.94 | .79  .68-.89 | .82  .72-.92 | .71  .58-.83 | .60  .47-.74 | .43  .30-.57 | .55  .41-.70 |
|  | GAD 37°C |  |  |  |  |  | .95  .89-1.0 | .90  .82-.97 | .88  .80-.96 | .87  .78-.95 | .80  .70-.91 | .80  .70-.91 | .72  .60-.84 | .59  0.45-0.72 | .45  .31-.58 | .54  .39-.68 |
|  | GAD 4°C |  |  |  |  |  |  | .88  .80-.96 | .84  .74-.93 | .85  .76-.94 | .79  .68-.89 | .82  .72-.92 | .71  .58-.83 | .60  .47-.74 | .43  .30-.57 | .58  .44-.72 |
|  | RAD 22°C |  |  |  |  |  |  |  | .92  .85-.98 | .90  .83-.97 | .77  .66-.88 | .77  .66-.88 | .73  .60-.84 | .62  .48-.75 | .42  .29-.55 | .60  .46-.74 |
|  | RAD 37°C |  |  |  |  |  |  |  |  | .95  .89-1.0 | .79  .68-.89 | .75  .64-.87 | .77  .66-.88 | .61  .47-.74 | .46  .32-.59 | .55  .41-.70 |
|  | RAD 4°C |  |  |  |  |  |  |  |  |  | .74  .62-.85 | .74  .62-.86 | .72  .60-.84 | .62  .48-.75 | .42  .29-.55 | .54  .39-.68 |
| **GEL KIT** | RAD 22°C |  |  |  |  |  |  |  |  |  |  | .93  .87-.99 | .78  .67-.89 | .66  .53-.78 | .52  .37-.66 | .57  .43-.71 |
|  | RAD 22°C washed |  |  |  |  |  |  |  |  |  |  |  | .75  .64-.87 | .72  .60-.83 | .47  .33-.61 | .60  .46-.74 |
| **GEL LAB** | GAD 22°C |  |  |  |  |  |  |  |  |  |  |  |  | .64  .52-.76 | .43  .29-.58 | .58  .45-.72 |
|  | GAD 22°C washed |  |  |  |  |  |  |  |  |  |  |  |  |  | .29  .18-.40 | .69  .57-.82 |
|  | RAD 22°C |  |  |  |  |  |  |  |  |  |  |  |  |  |  | .34  .21-.47 |

**Supplementary Table S11** Comparison of six direct antiglobulin test (DAT) results in 126 dogs suspected to have immune-mediated hemolytic anemia (IMHA). The Cohen’s kappa (κ) values and a two-sided confidence intervals (95%) is reported for each pair of methods used

DAT methods: STRIP KIT: in-clinic immunochromatographic strip kit; FLOW: flow cytometry (n = 69); CAPIL: microcapillary tube; MICRO: microtiter plate; GEL KIT: in-clinic gel minitube kit; GEL LAB: neutral gel column card with added antiglobulin
Reagents: GAD: goat anti-dog IgG, IgM, and C3; RAD: rabbit anti-dog IgG, IgM, and C3, GAD*: FITC-marked goat anti-dog IgG (H+L)
Groups were compared by Cohen’s kappa, κ = .29 to .95, p < .001. Interpretation of the κ values: Green: very good agreement (≥ .81); yellow: good agreement (≥ .61); blue: moderate agreement (≥ .41); white: fair agreement (≥ .21), as per Landis and Koch [1] and adapted by Brennan and Silman [2]

**Supplementary Table S12** Comparison of six direct antiglobulin test (DAT) results in 126 dogs suspected to have immune-mediated hemolytic anemia (IMHA) and 28 healthy dogs. The Cohen’s kappa (κ) values and two-sided confidence intervals (95%) are reported for each pair of methods used

| **DAT Method** | Antiglobulin  Temperature  Washing | **FLOW** | **CAPIL** | | **MICRO** | | | | | | | **GEL KIT** | | | **GEL LAB** | | | |
| --- | --- | --- | --- | --- | --- | --- | --- | --- | --- | --- | --- | --- | --- | --- | --- | --- | --- | --- |
|  |  | GAD*  22°C | GAD  22°C | RAD  22°C | GAD 22°C | GAD 37°C | GAD  4°C | RAD  22°C | RAD  37°C | RAD  4°C | RAD  22°C | | RAD  22°C washed | GAD  22°C | | GAD  22°C washed | RAD  22°C | RAD  22°C  washed |
| **STRIP KIT** | RAD 22°C | .73  .59-.88 | .63  .52-.75 | .78  .68-.87 | .81  .72-.90 | .83  .74-.91 | .79  .64-.88 | .80  .71-.89 | .84  .76-.92 | .80  .71-.89 | .80  .71-.89 | | .78  .68-.87 | .76  .66-.86 | | .60  .48-.71 | .58  .45-.70 | .56  .43-.68 |
| **FLOW** | GAD 22°C |  | .55  .39-.71 | .67  .52-.82 | .67  .52-.82 | .67  .52-.82 | .63  .48-.79 | .67  .52-.82 | .72  .57-.86 | .65  .50-.81 | .72  .57-.86 | | .67  .52-.82 | .72  .57-.86 | | .49  .33-.65 | .63  .46-.80 | .49  .32-.65 |
| **CAPIL** | GAD 22°C |  |  | .79  .70-.89 | .73  .62-.83 | .71  .60-.82 | .75  .07-.86 | .74  .63-.85 | .70  .59-.81 | .74  .63-.85 | .75  .64-.85 | | .74  .64-.85 | .68  .57-.79 | | .75  .64-.86 | .41  .30-.51 | .69  .57-.81 |
|  | RAD 22°C |  |  |  | .85  .77-.93 | .89  .82-.96 | .88  .80-.95 | .86  .78-.94 | .85  .77-.93 | .84  .75-.92 | .84  .75-.92 | | .84  .75-.92 | .75  .64-.85 | | .73  .62-.83 | .53  .42-.65 | .66  .54-.78 |
| **MICRO** | GAD 22°C |  |  |  |  | .96  .91-1.0 | .94  .89-.99 | .93  .87-.99 | .89  .82-.96 | .88  .80-.95 | .83  .74-.91 | | .85  .77-.93 | .76  .66-.86 | | .66  .54-.78 | .52  .41-.64 | .62  .50-.75 |
|  | GAD 37°C |  |  |  |  |  | .96  .91-1.0 | .92  .85-.98 | .90  .84-.97 | .89  .82-.96 | .84  .75-.92 | | .84  .75-.92 | .77  .67-.87 | | .64  .52-.76 | .53  .42-.65 | .61  .48-.73 |
|  | GAD 4°C |  |  |  |  |  |  | .90  .84-.97 | .86  .78-.94 | .88  .80-.95 | .83  .74-.91 | | .85  .77-.93 | .76  .66-.86 | | .66  .54-.78 | .52  .41-.64 | .65  .53-.77 |
|  | RAD 22°C |  |  |  |  |  |  |  | .93  .87-.99 | .92  .85-.98 | .81  .72-.90 | | .81  .72-.90 | .77  .67-.87 | | .67  .55-.79 | .51  .40-.63 | .66  .54-.78 |
|  | RAD 37°C |  |  |  |  |  |  |  |  | .96  .91-1.0 | .83  .74-.91 | | .80  .70-.89 | .81  .72-.90 | | .66  .54-.78 | .54  .43-.66 | .62  .50-.75 |
|  | RAD 4°C |  |  |  |  |  |  |  |  |  | .79  .69-.88 | | .78  .69-.88 | .77  .67-.87 | | .67  .55-.79 | .51  .40-.62 | .61  .48-.73 |
| **GEL KIT** | RAD 22°C |  |  |  |  |  |  |  |  |  |  | | .94  .89-.99 | .83  .74-.91 | | .71  .60-.81 | .60  .48-.71 | .64  .52-.76 |
|  | RAD 22°C washed |  |  |  |  |  |  |  |  |  |  | |  | .80  .70-.89 | | .75  .65-.86 | .56  .44-.67 | .66  .55-.78 |
| **GEL LAB** | GAD 22°C |  |  |  |  |  |  |  |  |  |  | |  |  | | .69  .58-.80 | .54  .42-.66 | .65  .54-.77 |
|  | GAD 22°C washed |  |  |  |  |  |  |  |  |  |  | |  |  | |  | .38  .27-.48 | .73  .62-.84 |
|  | RAD 22°C |  |  |  |  |  |  |  |  |  |  | |  |  | |  |  | .44  .33-.55 |

DAT methods: STRIP KIT: in-clinic immunochromatographic strip kit; FLOW: flow cytometry (n = 69); CAPIL: microcapillary tube; MICRO: microtiter plate; GEL KIT: in-clinic gel minitube kit; GEL LAB: neutral gel column card with added antiglobulin
Reagents: GAD: goat anti-dog IgG, IgM, and C3; RAD: rabbit anti-dog IgG, IgM, and C3, GAD*: FITC-marked goat anti-dog IgG (H+L)
Groups were compared by Cohen’s kappa, κ = .38 to .96, p < .001. Interpretation of the κ value: Green: very good agreement (≥ .81); yellow: good agreement (≥ .61); blue: moderate agreement (≥ .41); white: fair agreement (≥ .21), as per Landis and Koch [1] and adapted by Brennan and Silman [2]

**Supplement Table S13** Cohen’s kappa (κ) value of spherocytosis, and macroscopic in tube agglutination and macroscopic saline agglutination test (SAT) results at two dilutions compared to the microtiter plate DAT method with goat-anti dog IgG, IgM, and C3 at 22°C

|  | **In Tube Agglutination** |  | **SAT 1:1** | |  | **SAT 1:4** |  | **Agglutination After Washing** | **Spherocytosis** |
| --- | --- | --- | --- | --- | --- | --- | --- | --- | --- |
|  |  |  |  |  | |  |  |  |  |
| Κ value | .27 |  | .39 |  | | .39 |  | .05 | .80 |
| 95% CI | .12 - .42 |  | .23 - .54 |  | | .23 - .54 |  | .01 - .11 | .69 - .90 |
|  |  |  |  |  | |  |  |  |  |
|  |  |  |  |  | |  |  |  |  |
| Sensitivity | 43.3 |  | 56.7 |  | | 56.7 |  | 6.0 | 87.1 |
| Specificity | 84.7 |  | 83.1 |  | | 83.1 |  | 100 | 93.1 |
| p value | .001 |  | < .001 |  | | < .001 |  | .056 | < .001 |
|  |  |  |  |  | |  |  |  |  |

95% CI: 95% confidence interval
Groups were compared by Cohen’s kappa (κ) test. Interpretation of the Κ values: very good agreement (≥ .81); good agreement (≥ .61); moderate agreement (≥ .41); fair agreement (≥ .21), as per Landis and Koch [1] and adapted by Brennan and Silman [2]


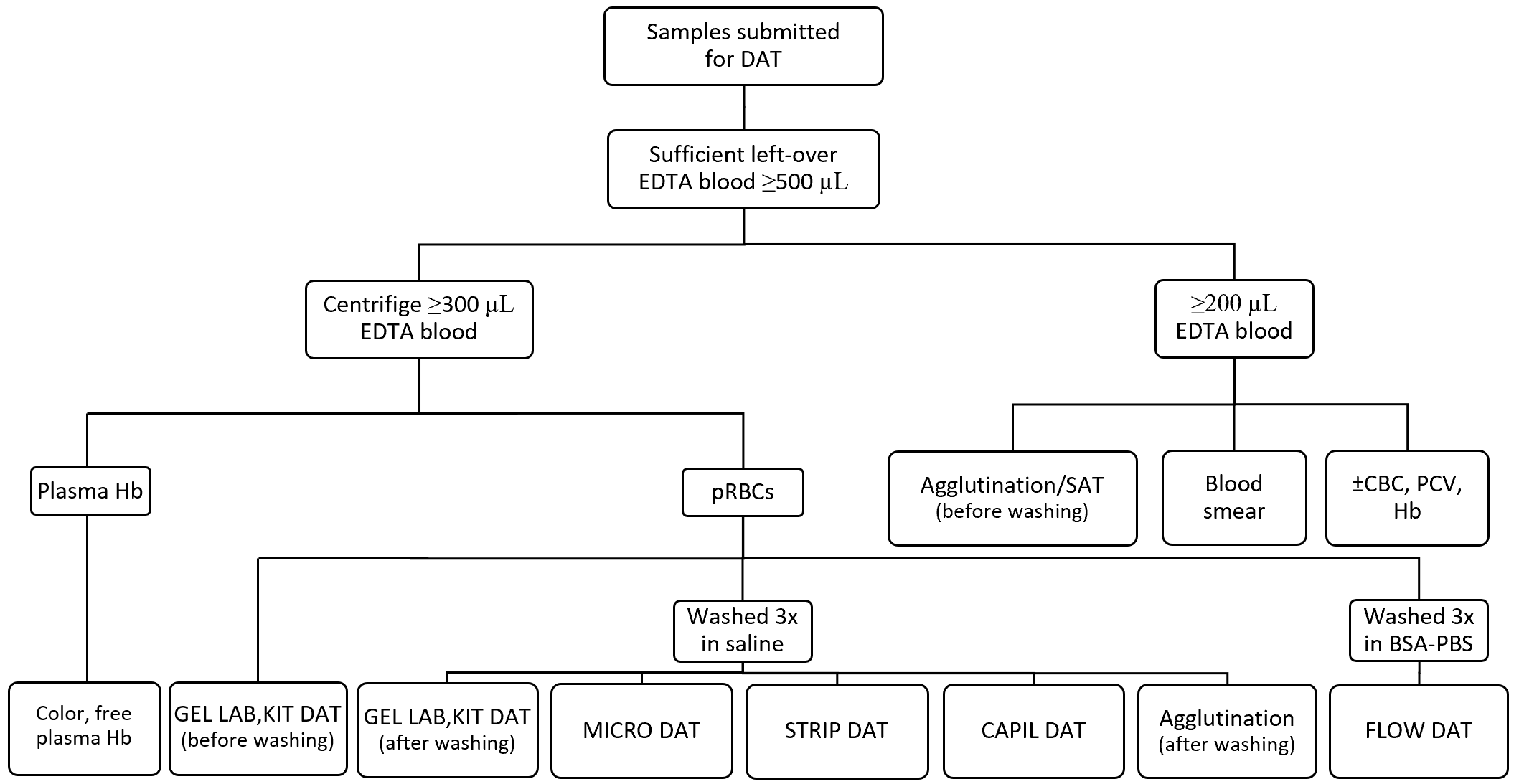
**Supplementary Figure S1** Flow chart of laboratory tests and six different direct antiglobulin test (DAT) methods performed with blood samples from dogs suspected to have immune-mediated hemolytic anemia

Hb: hemoglobin; pRBCs: packed red blood cells; CBC: complete blood count; PCV: packed cell volume; BSA-PBS: bovine serum albumin-phosphate-buffered saline; SAT: saline agglutination test
**DAT methods:** STRIP KIT: in-clinic immunochromatographic strip kit; FLOW: flow cytometry; CAPIL: microcapillary tube; GEL KIT: in-clinic gel minitube kit; GEL LAB: gel column; neutral gel column card with added antiglobulin
In addition, blood samples were identically analyzed from healthy dogs as negative and positive controls.

**Supplementary Figure S2** Severity of anemia, reticulocytosis, hyperbilirubinemia, and free plasma hemoglobin concentration compared to microtiter plate direct antiglobulin test (MICRO DAT) results in dogs suspected to have immune-mediated hemolytic anemia


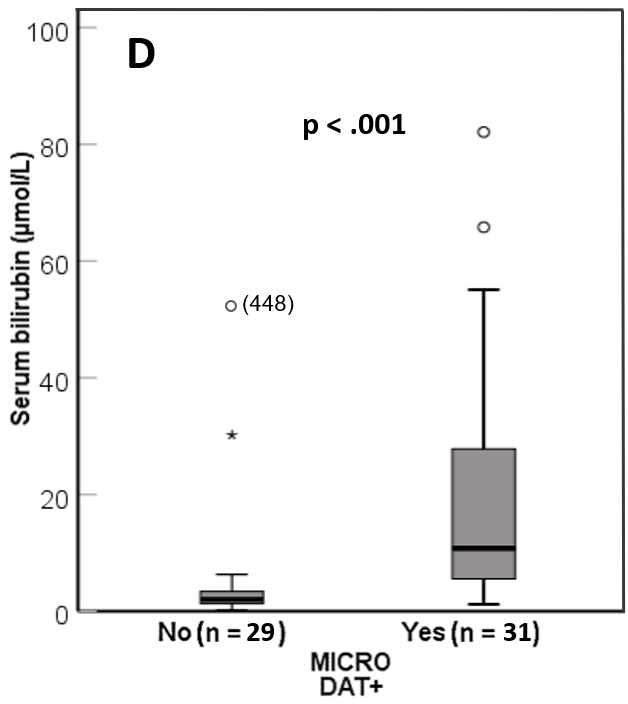

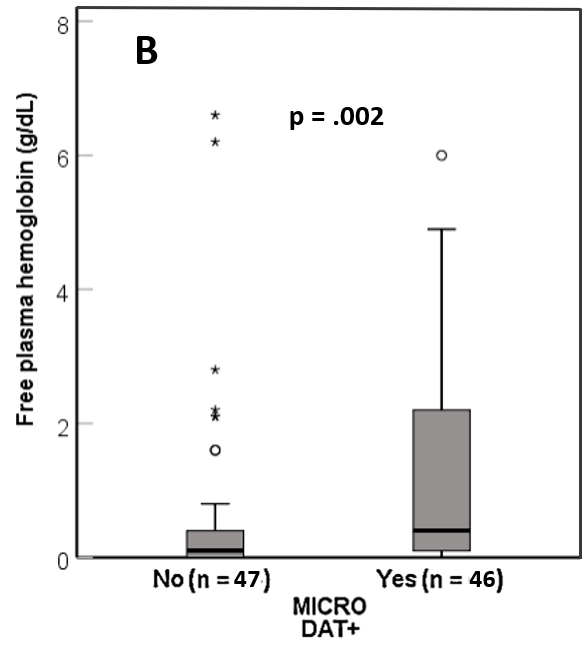

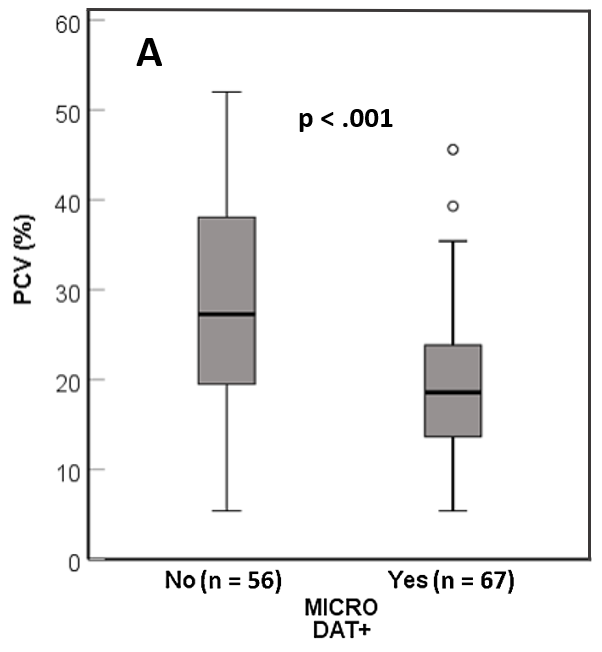


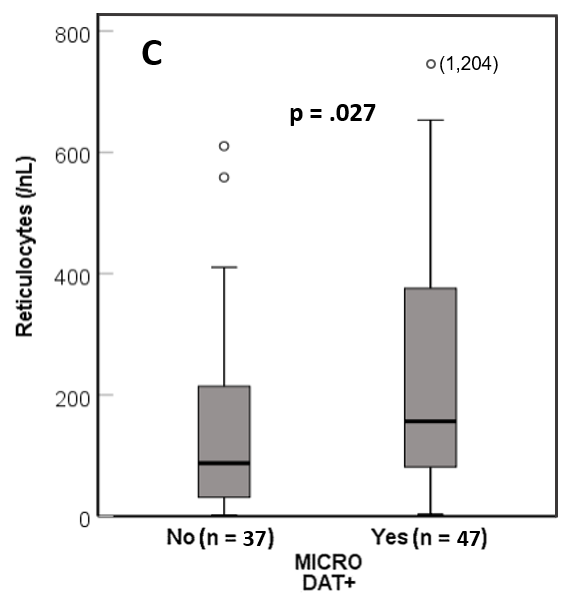
PCV: packed cell volume; MICRO DAT+: microtiter plate direct antiglobulin test method with goat anti-dog IgG, IgM, and C3. Groups were compared by Student’s t test. Boxplot: The median is represented by the line within the box. Each upper and lower box ranges represent the first and third quartile. The upper and lower whiskers represent the minimum and maximum, excluding any outliers. Outliers are represented by white circles.

**Supplementary Figure S3** Severity of anemia, reticulocytosis, bilirubinemia, and free plasma hemoglobin concentration results grouped based on ≥4 DAT+ results in dogs suspected to have immune-mediated hemolytic anemia


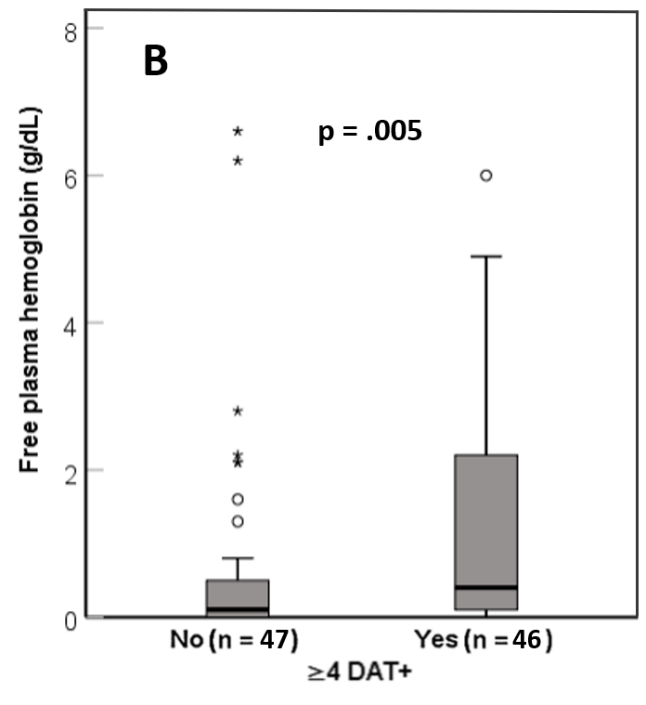

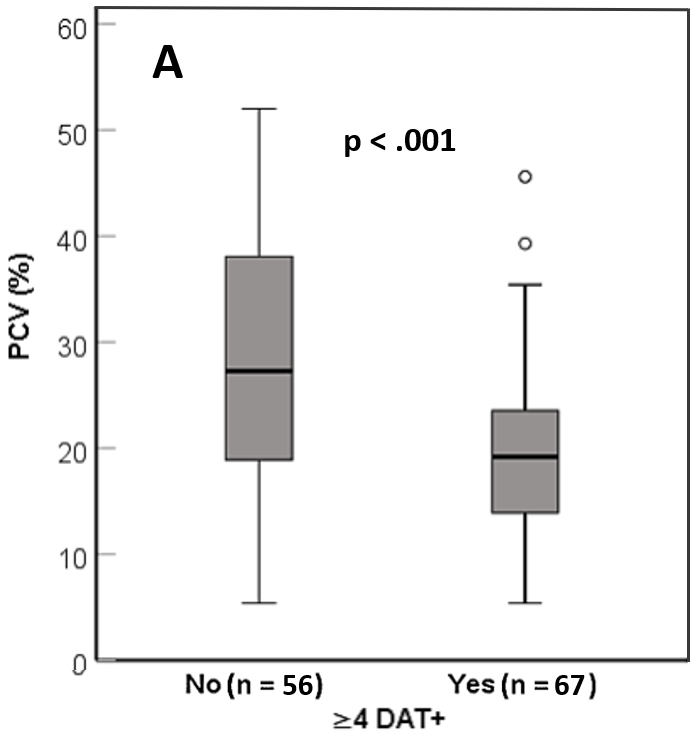


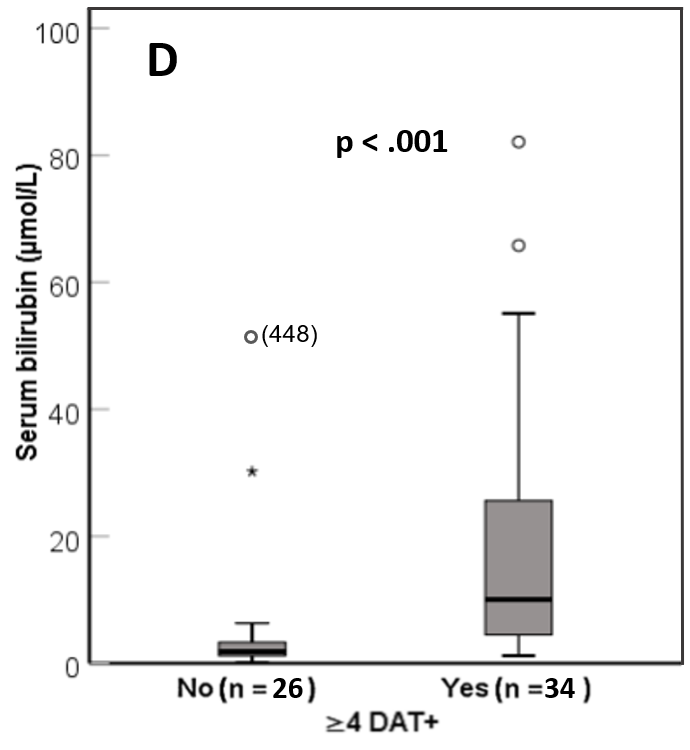


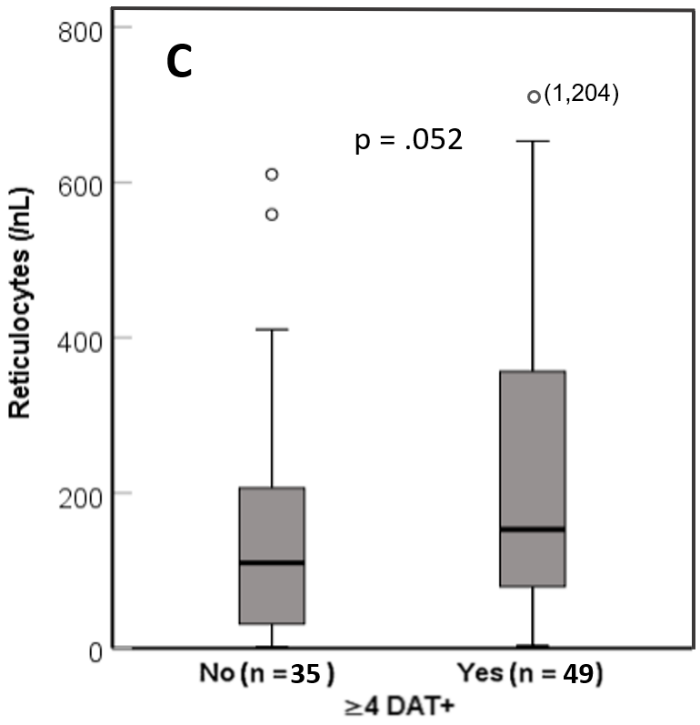
PCV: packed cell volume;. ≥4 DAT+: at least four different direct antiglobulin test (DAT) results were positive among the five

to six performed DATs. Groups were compared by Student’s t test. Boxplot: The median is represented by the line within the box. Each upper and lower box ranges represent the first and third quartile. The upper and lower whiskers represent the minimum and maximum, excluding any outliers. Outliers are represented by white circles.

1. Landis JR, Koch GG. An application of hierarchical kappa-type statistics in the assessment of majority agreement among multiple observers. Biometrics. 1977; https://doi.org/10.2307/252978.
2. Brennan P, Silman A. Statistical methods for assessing observer variability in clinical measures. BMJ. 1992; https://doi.org/10.1136/bmj.304.6840.1491.
